# Time trend of pancreatic cancer mortality in the Western Pacific Region: age-period-cohort analysis from 1990 to 2019 and forecasting for 2044

**DOI:** 10.1186/s12885-023-11369-1

**Published:** 2023-09-18

**Authors:** Wenkai Jiang, Caifei Xiang, Yan Du, Xiao Li, Xin Li, Wence Zhou

**Affiliations:** 1 The Second Clinical Medical College, Gansu Province, Lanzhou University, Cheng-Guan District, No. 222 Tianshui Road (South), Lanzhou City, 730030 China; 2 The First Clinical Medical College, Lanzhou University, Lanzhou, 730030 China; 3 Department of General Surgery, Lanzhou University Second Hospital, Lanzhou, 730030 China

**Keywords:** Pancreatic cancer, Western Pacific Region, Age-period-cohort model, Mortality, Forecasting

## Abstract

**Background:**

Pancreatic cancer poses a serious medical problem worldwide. Countries in the Western Pacific Region are facing public health challenges from cancer. This study assesses the time trends of pancreatic cancer mortality in the Western Pacific Region from 1990 to 2019 and predicts its trend to 2044.

**Methods:**

Mortality data were obtained from the Global Health Data Exchange. We used an age-period-cohort model to estimate age, period and birth cohort effects on pancreatic cancer mortality from 1990 to 2019 by calculating net drift, local drift, age-specific rate, period rate ratio, and cohort rate ratio. We also predict pancreatic cancer mortality to 2044 in Western Pacific countries.

**Results:**

Overall, there were 178,276 (95% uncertain interval: 157,771 to 198,636) pancreatic cancer deaths in the Western Pacific Region in 2019, accounting for 33.6% of all deaths due to pancreatic cancer worldwide. There were significant increases in pancreatic cancer disability-adjusted life years between 1990 and 2019 in the Western Pacific Region, mainly due to population growth and aging. Pancreatic cancer mortality increased with age. The period effect showed an increasing trend of mortality for both sexes over the study period. Compared to the reference period (2000 to 2004), the rate ratio was elevated in both males and females in the period of 2015 to 2019. There was an overall increasing rate ratio from early birth cohorts to recent cohorts. Deaths may continue to increase in the next 25 years in the ten countries, while most countries have seen their age-standardized rate forecasts fall.

**Conclusion:**

The mortality of pancreatic cancer is still high in the Western Pacific Region. Countries/territories should focus on pancreatic cancer prevention and early cancer screening in high-risk populations. Specific public health methods and policies aimed at reducing risk factors for pancreatic cancer are also needed.

**Supplementary Information:**

The online version contains supplementary material available at 10.1186/s12885-023-11369-1.

## Introduction

Pancreatic cancer is characterized by poor prognosis, with an overall 5-year survival rate of approximately 10% [1]. The global burden of pancreatic cancer has more than doubled over the past 25 years and now poses a serious medical problem worldwide [2]. The Western Pacific Region is one of the six World Health Organization (WHO) regions and is home to more than a quarter of the world’s population [3]. During the past few decades, the societies and economies of Western Pacific countries/territories have developed, leading to longer life expectancies [4]. The Western Pacific Regional Action Plan for the Prevention and Control of Noncommunicable Diseases 2014–2020 was endorsed by member states in 2013 to avoidable mortality due to noncommunicable diseases in the Western Pacific region [5]. However, the proportion of elderly individuals is growing faster than any other age group in the Western Pacific Region, which will lead to a high cancer burden.

Population aging is one of the important social characteristics in the Western Pacific Region [4]. Pancreatic cancer is typically a disease of older people, and age has been identified as an independent risk factor for pancreatic cancer patients [6]. Although there is ongoing research into pancreatic cancer burden, few studies have analyzed the time trends of pancreatic cancer mortality in Western Pacific countries/territories. Advances in the diagnosis and treatment of pancreatic cancer have been made over the past few years, but we still confront various health challenges. Under these circumstances, assessing, monitoring and comparing progress among pancreatic cancer patients in Western Pacific countries/territories are critical for cancer prevention and adjustment of strategies of treatment. Published studies have explored the trends in the disease burden of pancreatic cancer in some regions and countries [7–9]. However, studies focused on the age, period and birth cohort effects on pancreatic cancer mortality and predicting future trends remain lacking.

In this article, we conducted a systematic analysis based on the Global Burden of Disease Study (GBD) 2019 to explore the time trends of pancreatic cancer mortality in Western Pacific Region from 1990 to 2019, analyze the age, period, and cohort effects on mortality, and predict mortality to 2044, aiming to provide new insight into regional health.

## Methods

### Data source

The data were obtained from the Global Health Data Exchange available from https://ghdx.healthdata.org (accessed on January 15, 2023), including annual number, age-standardized rate (ASR) and their 95% uncertainty intervals (UIs). GBD 2019 provided 369 diseases and injuries and 87 risk factors for six WHO regions, 21 GBD regions and all countries/territories from 1990 to 2019 [10, 11]. In GBD 2019, pancreatic cancer was identified according to the International Classification of Diseases, version 10 as the following codes: C25-C25.9, Z85.07, contained primary malignancy of the pancreas (common and less common tumor types). More information about the methods and processing for quantifying data can be found in published studies [12, 13].

### Sociodemographic index (SDI)

The SDI is a comprehensive index that reflects the development of a country or region. It ranges from 0 to 1 and can be calculated by the overall fertility rate among females younger than 25 years old, the average education level of people aged 15 and older, and the per capita income [14]. The SDI values of countries/territories can be downloaded at https://ghdx.healthdata.org/record/ihme-data/gbd-2019-socio-demographic-index-sdi-1950-2019.

### Age-period-cohort model

The age-period-cohort model reflects the effects of age groups, periods of observation and birth cohorts on disease morbidity and mortality [15]. This approach aims to reveal the contribution of age-related social factors, technological factors and biological factors to disease trends [16]. The age effect refers to the impact of changes, including population aging, on pancreatic cancer mortality. The period effect refers to the change in risk of pancreatic cancer mortality of each age group caused by objective factors. The birth cohort effect refers to the effect of different levels of exposure to disease risk factors exposure on the mortality of different birth cohorts.

In a typical age-period-cohort model, the age and period intervals should be equal. Because GBD 2019 estimates are produced in 5-year age groups with data (15 to 19, 20 to 24, 25 to 29, ……, 90 to 94, 95 to 99), we arranged the pancreatic cancer deaths and global population data into successive 5-year periods (1990 to 1994, 1995 to 1999, 2000 to 2004, 2005 to 2009, 2010 to 2014, 2015 to 2019), with 2000 to 2004 (medium: 2002) as the reference period. Based on the equation (linear relationship between the three variables: birth cohort = period – age), twenty-two consecutive birth cohorts were also created (1895 to 1900, 1900 to 1905, 1905 to 1910, ……, 2000 to 2004) [16].

The estimated indexes were calculated and obtained from the age-period-cohort web tool (https://analysistools.cancer.gov/apc), including net drift, local drifts (estimated annual percentage change in each age group), longitudinal age curves (age-specific rate), period rate ratio (ratio of age-specific rates in a specific period relative to reference period) and cohort rate ratio (ratio of age-specific rates in a specific cohort relative to reference cohort). The methodological details of the age-period-cohort web tool are described in previous literature [17].

### Decomposition analysis

Decomposition analysis is used to identify the additive contribution of different effects in two populations to the difference in their overall value [18]. It will allow for a better understanding of the factors associated with the changes in the absolute number of age-related disease burdens during a period [19]. In this study, we constructed a decomposition analysis to evaluate changes in disability-adjusted life years (DALYs) by three factors: (1) population size, (2) aging population, and (3) epidemiologic changes.

### Data analysis

Data processing was performed in RStudio software (Version 4.2.2). The estimated annual percentage changes (EAPCs) and their 95% confidence intervals (CIs) were calculated to assess the mortality trend using linear regression analysis. The ASR showed an upward trend when the EAPC and its lower 95% CI were positive. In contrast, the ASR showed a downward trend when the EAPCs and their upper 95% CI were negative [20]. We performed classification analysis using the EAPCs of the ASR (1990 to 2019) and the ASR (in 2019). The 33rd and 66th percentiles (lower and upper terciles) were used to classify countries/territories into nine categories. The “Nordpred” package is used for predicting future deaths, which takes into account the data on global population projections and changing population structure. All rates are reported per 100,000 population. Visualizations were performed by the “ggplot2” package.

## Results

### An overview of burden

**Fig. 1:**
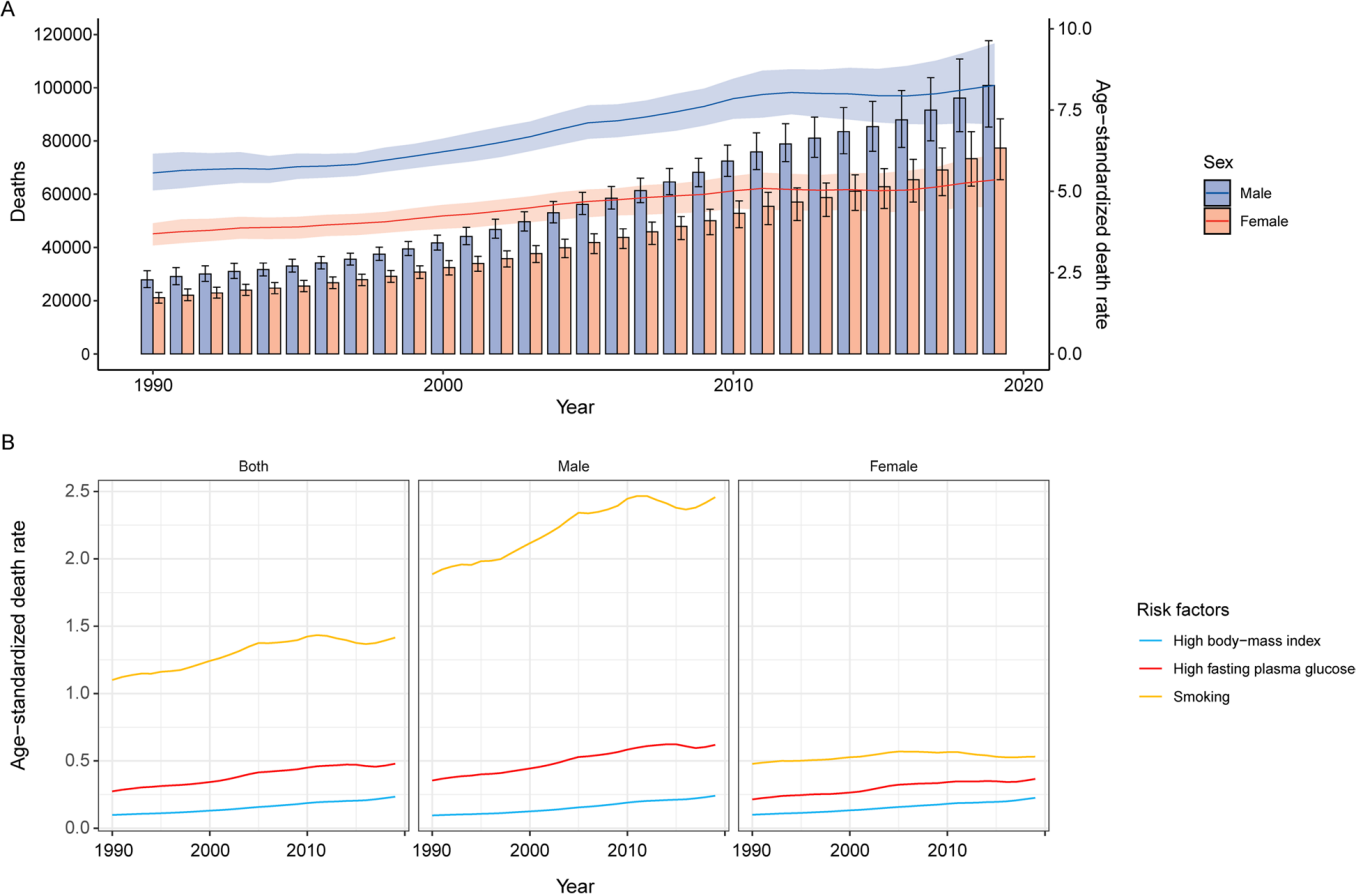
Time trends of pancreatic cancer mortality in the Western Pacific Region, 1990 to 2019. **A** Counts and age-standardized rates of pancreatic cancer mortality by sex, 1990 to 2019. **B** Age-standardized death rate for pancreatic cancer attributable to smoking, high fasting plasma glucose and high body mass index by sex, 1990 to 2019

**Fig. 2:**
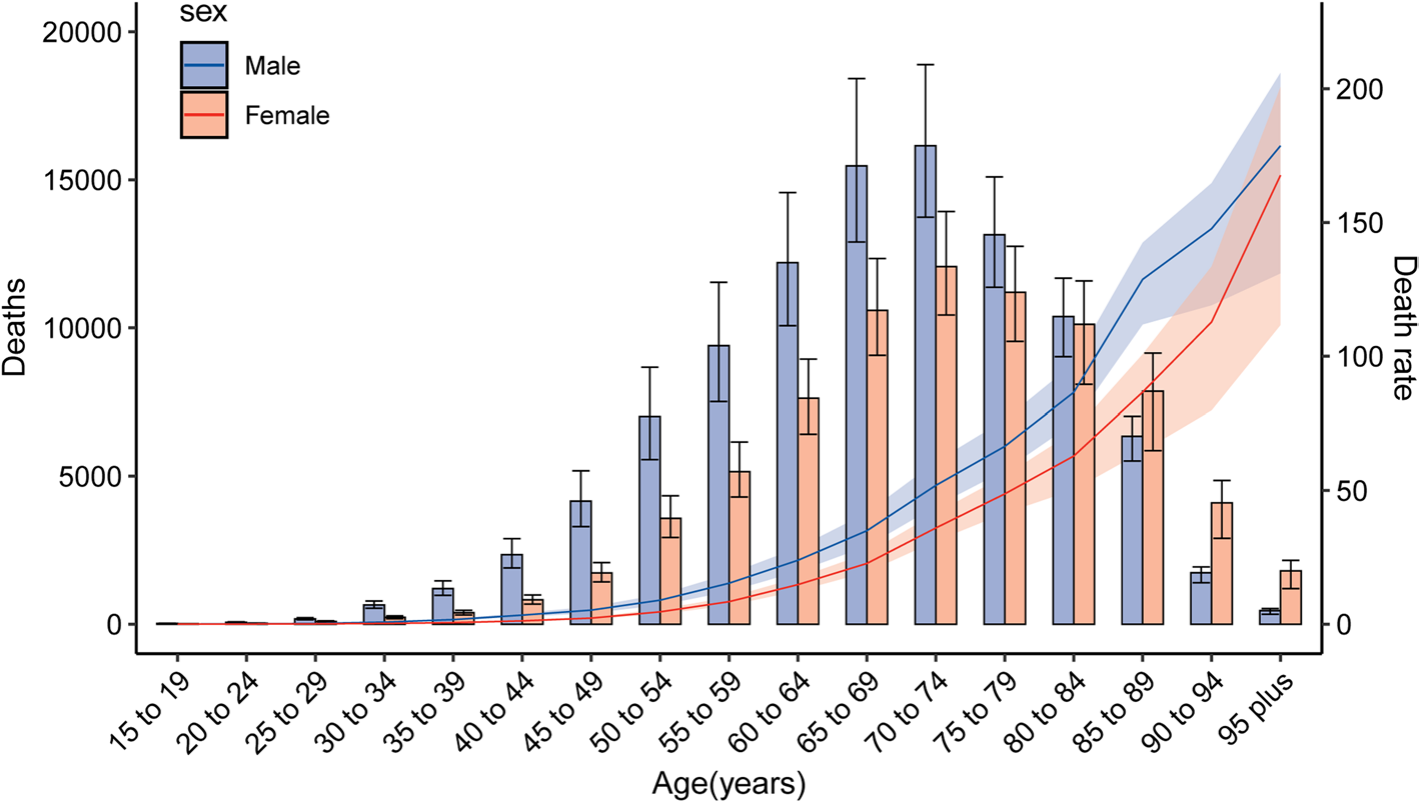
Age-specific counts and rates of deaths of pancreatic cancer by sex in Western Pacific Region, 2019

Table 1 shows the mortality for pancreatic cancer globally and in the Western Pacific Region in 2019 and its change trend from 1990 to 2019. Overall, there were 178,276 (95% UI: 157,771 to 198,636) deaths due to pancreatic cancer in the Western Pacific Region in 2019, accounting for 33.6% of all pancreatic cancer deaths worldwide. The age-standardized death rate (ASDR) was 6.71 (95% UI: 5.92 to 7.45) per 100,000 population in 2019, which was nearly 1.4-fold higher than that in 1990 (4.54, 95% UI: 4.19 to 4.87). During the past 30 years, males always had higher number of deaths and ASDRs than females (Fig. 1A). The ASDR in the Western Pacific Region increased from 1990 to 2019 at an EAPC of 1.5 (95% CI: 1.39 to 1.62) and at a percent change of 47.8% (30.3% to 67.2%), which is higher than the level observed globally. The number of pancreatic cancer deaths in the Western Pacific Region peaked at the ages of 70 to 74 years for both sexes in 2019. The number of deaths is lower in females younger than 85 years than in males in the same age group, whereas the number is higher in females than in males in age groups of 85 years and older. Age-specific death rates increased with increasing age for both sexes; death rates for males were always higher than those for females in all age groups (Fig. 2).

**Table 1:** Mortality for pancreatic cancer in Western Pacific Region in 2019 and their change trend from 1990 to 2019

	**Global**	**Western Pacific Region**
**2019**		
Total mortality		
Number of deaths	531,107 (491,948 to 566,537)	178,276 (157,771 to 198,636)
ASDR	6.62 (6.11 to 7.06)	6.71 (5.92 to 7.45)
Mortality due to smoking		
Number of deaths	113,384 (98,830 to 128,466)	38,359 (31,806 to 45,750)
ASDR	1.4 (1.22 to 1.59)	1.42 (1.18 to 1.67)
Mortality due to high fasting plasma glucose		
Number of deaths	48,358 (11,540 to 103,691)	12,738 (2,881 to 28,589)
ASDR	0.61 (0.14 to 1.30)	0.48 (0.11 to 1.08)
Mortality due to high body-mass index		
Number of deaths	31,921 (11,964 to 59,676)	6,312 (1,812 to 14,033)
ASDR	0.40 (0.15 to 0.74)	0.23 (0.07 to 0.52)
**1990 to 2019**		
Total mortality (age-standardized)		
Percentage change, 1990 to 2010	18.6% (14.2% to 23.2%)	39.7% (27.9% to 51.8%)
Percentage change, 2010 to 2019	4.5% (-0.6% to 9.4%)	5.8% (-5.1% to 18.1%)
Percentage change, 1990 to 2019	23.9% (16.4% to 32.4%)	47.8% (30.3% to 67.2%)
EAPC, 1990 to 2019	0.77 (0.73 to 0.81)	1.5 (1.39 to 1.62)

*ASDR* age-standardized death rate, *EAPC* estimated annual percentage change

GBD 2019 estimated that smoking, high fasting plasma glucose and high body mass index (BMI) are the three main risk factors for pancreatic cancer. The age-standardized proportions of all pancreatic cancer deaths that were attributable to these three risks in the Western Pacific Region in 2019 were 21.5%, 7.1% and 3.5%, respectively. From 1990 to 2019, the ASDRs of the three risk factors all showed upward trends in both sexes, and the ASDRs for all three risk factors were higher for males than for females (Fig. 1B). The fractions of pancreatic cancer age-specific deaths attributable to different risk factors by age group for males and females in 2019 are shown in Fig. S1.

Among the countries/territories in this study, China (117,374) had the highest number of deaths in 2019, followed by Japan (37,462) and the Republic of Korea (7,303) (Table 2). The highest and lowest ASDRs were observed in Palau (11.95) and Papua New Guinea (1.81), respectively (Tables S1-S3). From 1990 to 2019, only Samoa (EAPC = -0.38, 95% CI: -0.61 to -0.15) and Republic of Korea (EAPC = -0.33, 95% CI: -0.57 to -0.09) showed downward trends in the ASDR. Vietnam had seen the fastest growth in the ASDR of pancreatic cancer (EAPC = 3.76, 95% CI: 3.54 to 3.98). The deaths and ASDRs of pancreatic cancer attributable to smoking, high fasting plasma glucose, and high BMI in 1990 and 2019 are shown in Tables S4-S6. The fractions of pancreatic cancer age-standardized deaths attributable to three risk factors among countries/territories for males and females in 2019 are shown in Fig. S2.

**Table 2:** Counts and Age-standardized rate of pancreatic cancer mortality in Western Pacific countries/territories and their change trends from 1990 to 2019

Location	Deaths in 1990	ASDR in 1990	Deaths in 2019	ASDR in 2019	EAPC of ASDR
American Samoa	1 (1–1)	3.26 (2.72–3.77)	2 (2–3)	5.19 (4.34–6.16)	1.64 (1.23–2.06)
Australia	1476 (1404–1530)	7.55 (7.15–7.84)	3561 (3177–3941)	8.26 (7.45–9.08)	0.33 (0.26–0.4)
Brunei Darussalam	6 (5–7)	6.62 (5.64–7.7)	23 (20–26)	9.3 (8.06–10.61)	1.55 (1.38–1.73)
Cambodia	96 (71–124)	2.23 (1.66–2.89)	418 (334–503)	3.75 (3.01–4.41)	1.92 (1.81–2.04)
China	27,104 (23,604–30844)	3.34 (2.93–3.76)	117,374 (99,863–136,453)	5.99 (5.12–6.93)	2.25 (2.06–2.44)
Cook Islands	0 (0–1)	3.8 (3.09–4.56)	1 (1–1)	4.95 (4.17–5.85)	0.62 (0.46–0.78)
Fiji	9 (7–11)	2.74 (2.23–3.33)	30 (23–37)	4.34 (3.43–5.37)	1.51 (1.44–1.58)
Guam	3 (2–3)	4.28 (3.59–4.99)	10 (8–12)	5.26 (4.36–6.25)	0.98 (0.75–1.2)
Japan	15,361 (14,606–15775)	9.17 (8.68–9.43)	37,462 (31,496–40,785)	9.6 (8.4–10.26)	0.34 (0.24–0.44)
Kiribati	1 (1–1)	2.37 (1.93–2.8)	2 (1–2)	3 (2.3–3.9)	0.33 (0.01–0.64)
Lao People's Democratic Republic	49 (34–68)	2.4 (1.71–3.27)	143 (111–182)	3.52 (2.78–4.38)	1.29 (1.2–1.38)
Malaysia	167 (142–195)	1.95 (1.65–2.32)	949 (736–1194)	3.8 (2.98–4.76)	2.44 (2.05–2.83)
Marshall Islands	0 (0–0)	2.42 (2.02–2.87)	1 (1–2)	4.01 (3.01–5.21)	1.72 (1.64–1.81)
Micronesia (Federated States of)	1 (1–2)	3.24 (2.61–4)	3 (2–5)	5.31 (3.75–7.09)	1.53 (1.29–1.77)
Mongolia	32 (26–39)	3.23 (2.63–3.85)	138 (106–177)	6.28 (4.99–7.86)	2.28 (2.11–2.45)
Nauru	0 (0–0)	4.35 (3.29–5.7)	0 (0–0)	6.08 (4.26–8.17)	0.73 (0.53–0.92)
New Zealand	283 (265–302)	7.16 (6.7–7.63)	597 (540–653)	7.46 (6.8–8.11)	0.22 (0.08–0.37)
Niue	0 (0–0)	3.87 (3.04–4.71)	0 (0–0)	6.4 (4.89–8.14)	1.66 (1.47–1.85)
Northern Mariana Islands	1 (0–1)	3.55 (2.96–4.35)	4 (3–4)	7.75 (6.62–8.96)	3.39 (2.74–4.04)
Palau	1 (1–1)	8.38 (6.44–10.81)	2 (2–3)	11.95 (9.23–14.96)	1.1 (0.97–1.24)
Papua New Guinea	22 (14–33)	1.26 (0.8–1.89)	79 (55–113)	1.81 (1.28–2.56)	1.15 (1.07–1.23)
Philippines	959 (849–1078)	3.39 (3.04–3.77)	3250 (2654–3958)	4.41 (3.64–5.32)	0.53 (0.23–0.82)
Republic of Korea	2198 (2098–2321)	7.74 (7.33–8.19)	7303 (6519–8150)	8.25 (7.34–9.2)	-0.33 (-0.57–-0.09)
Samoa	4 (3–4)	4.26 (3.54–5.12)	6 (5–8)	4.4 (3.57–5.59)	-0.38 (-0.61–-0.15)
Singapore	113 (105–122)	5.44 (5.03–5.84)	425 (380–466)	5.59 (4.97–6.14)	0.33 (0.2–0.46)
Solomon Islands	3 (2–4)	1.87 (1.36–2.51)	9 (6–13)	3.07 (2.18–4.03)	1.53 (1.27–1.78)
Tokelau	0 (0–0)	2.65 (1.98–3.38)	0 (0–0)	4.46 (3.15–5.76)	1.79 (1.69–1.89)
Tonga	2 (1–2)	2.96 (2.28–3.73)	3 (3–5)	4.49 (3.29–5.87)	0.97 (0.51–1.43)
Tuvalu	0 (0–0)	2.62 (2.1–3.17)	0 (0–1)	4.02 (2.98–5.37)	1.24 (1.06–1.41)
Vanuatu	1 (1–2)	1.91 (1.39–2.62)	5 (4–7)	3.11 (2.42–4.01)	1.59 (1.49–1.7)
Viet Nam	706 (583–835)	1.82 (1.5–2.14)	3924 (3070–4960)	4.53 (3.57–5.68)	3.76 (3.54–3.98)

*ASDR* age-standardized death rate, *EAPC* estimated annual percentage change

### Classification and decomposition analysis

**Fig. 3:**
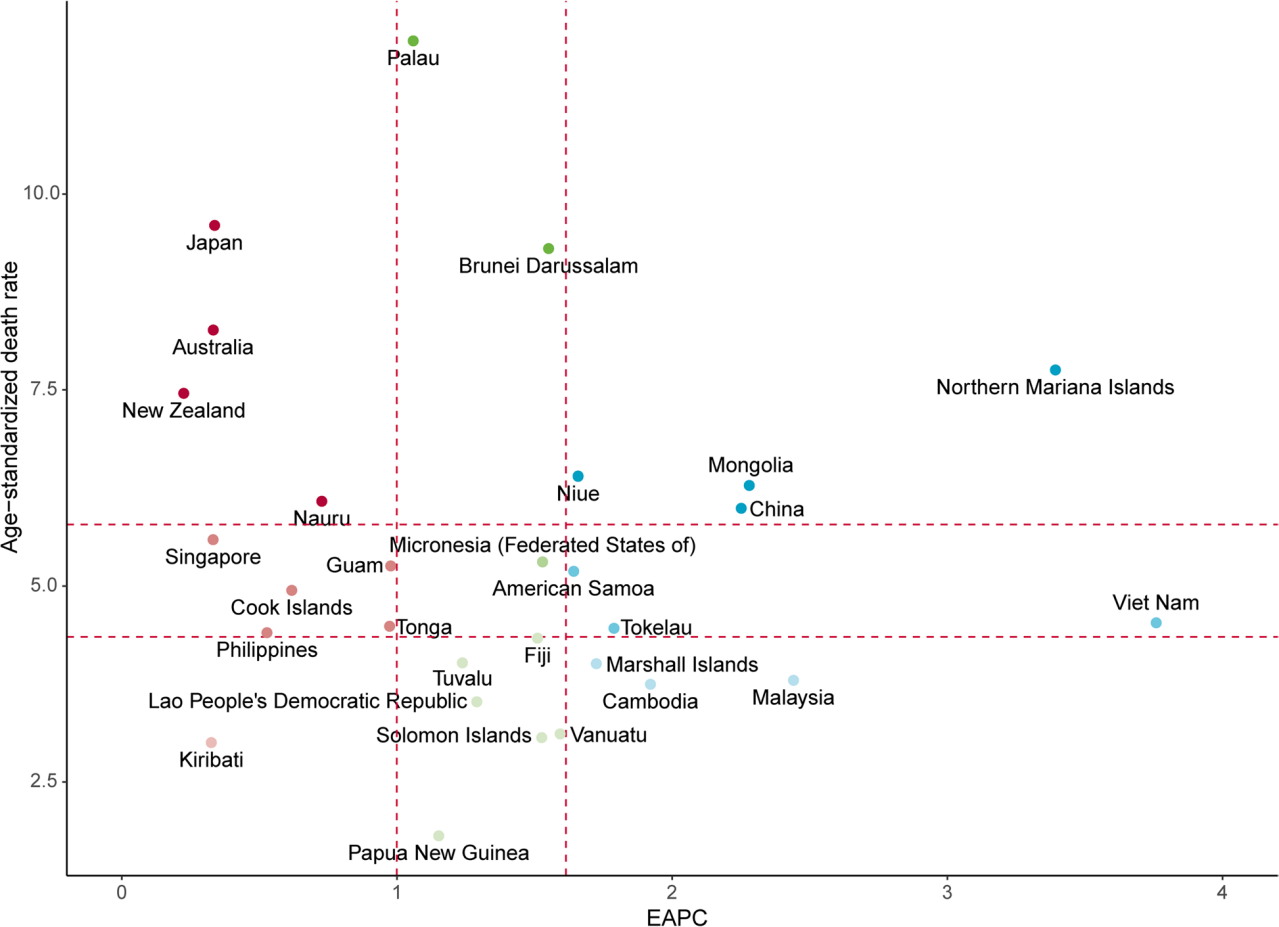
Classification analysis of Western Pacific countries by estimated annual percentage changes (1990 to 2019) and age-standardized death rate (2019) of pancreatic cancer mortality. EAPC: estimated annual percentage change

To understand the burden differences between countries, we performed a quadrant analysis using the EAPCs of the ASDR (1990 to 2019, only contained countries with EAPC > 0) and the ASDR (in 2019) (Fig. 3). Ten countries/territories were in the upper tercile of the distribution, which represents that they had the highest disease burden in 2019. Among them, the Northern Mariana Islands, Mongolia, Niue and China are in the upper tercile of the distribution of the EAPC, indicating that these countries have the greatest increase in the mortality of pancreatic cancer. Although the ASDRs in Japan, Australia and New Zealand increased slightly, these countries still had the highest burden in 2019. Kiribati is the only country located in the lower tercile of the distribution in the ASDR and EAPC combined.

**Fig. 4:**
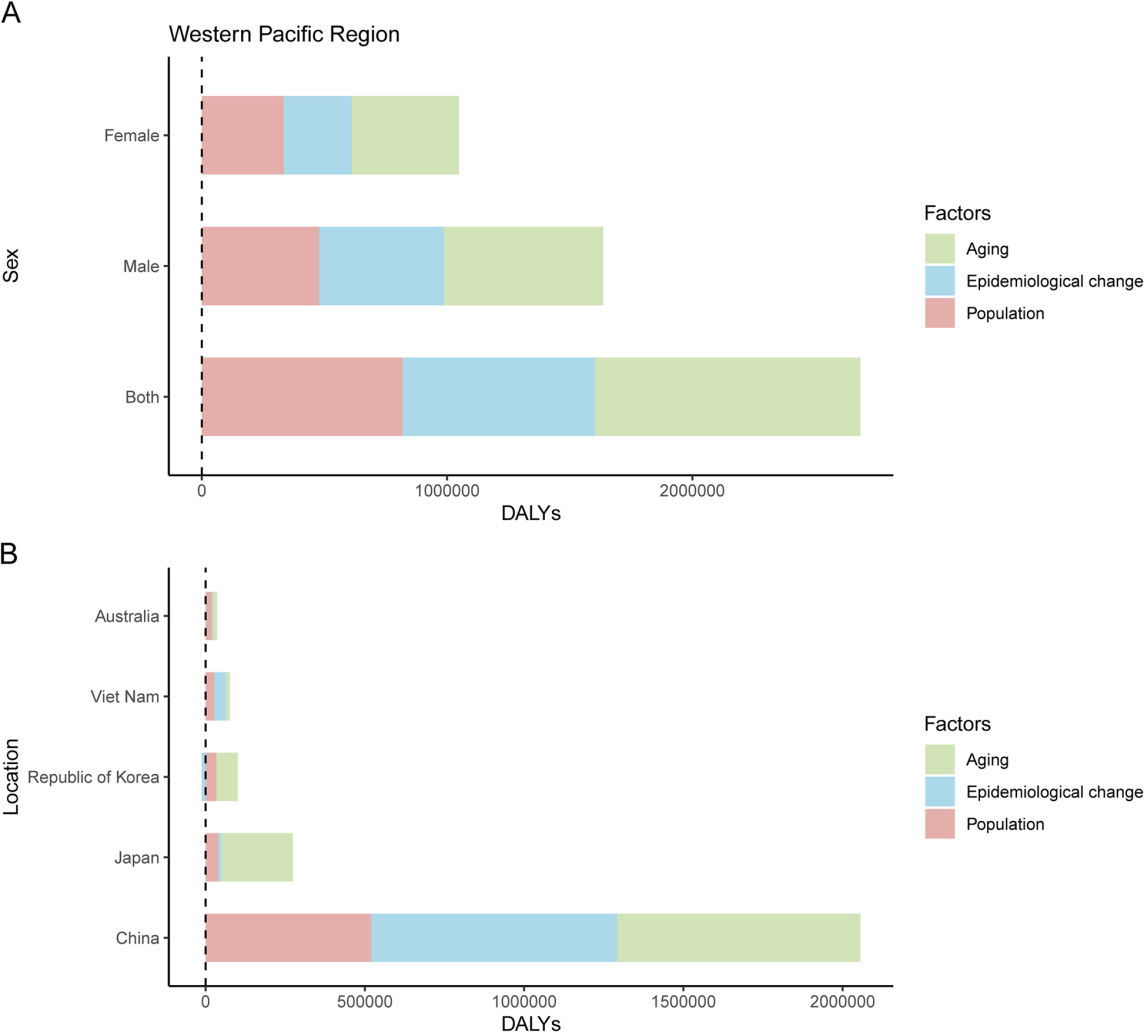
Decomposition analysis of age-related DALYs of pancreatic cancer in the Western Pacific Region, between 1990 and 2019. **A** Decomposition analysis by sex in the Western Pacific Region. **B** Decomposition analysis of five Western Pacific countries. DALYs: disability adjusted life years

We developed a decomposition analysis of pancreatic cancer DALYs by population size, age structure, and epidemiologic changes. As shown in Fig. 4A, there were significant increases in pancreatic cancer DALYs in the Western Pacific Region for both sexes. Population growth, aging and epidemiologic changes contributed 40.3%, 30.5% and 29.2% in both sexes, respectively, to the increased DALYs between 1990 and 2019. In the five countries with the highest deaths, the contribution of aging to the overall DALY increase was most pronounced in Japan (82.1%). Population growth in Australia accounted for the highest proportion (55.1%) of the increase in the five countries (Fig. 4B). Decomposition analysis of DALYs attributable to three risk factors is shown in Figs. S3-S5.

### Time trends in pancreatic cancer mortality in different age groups

**Fig. 5:**
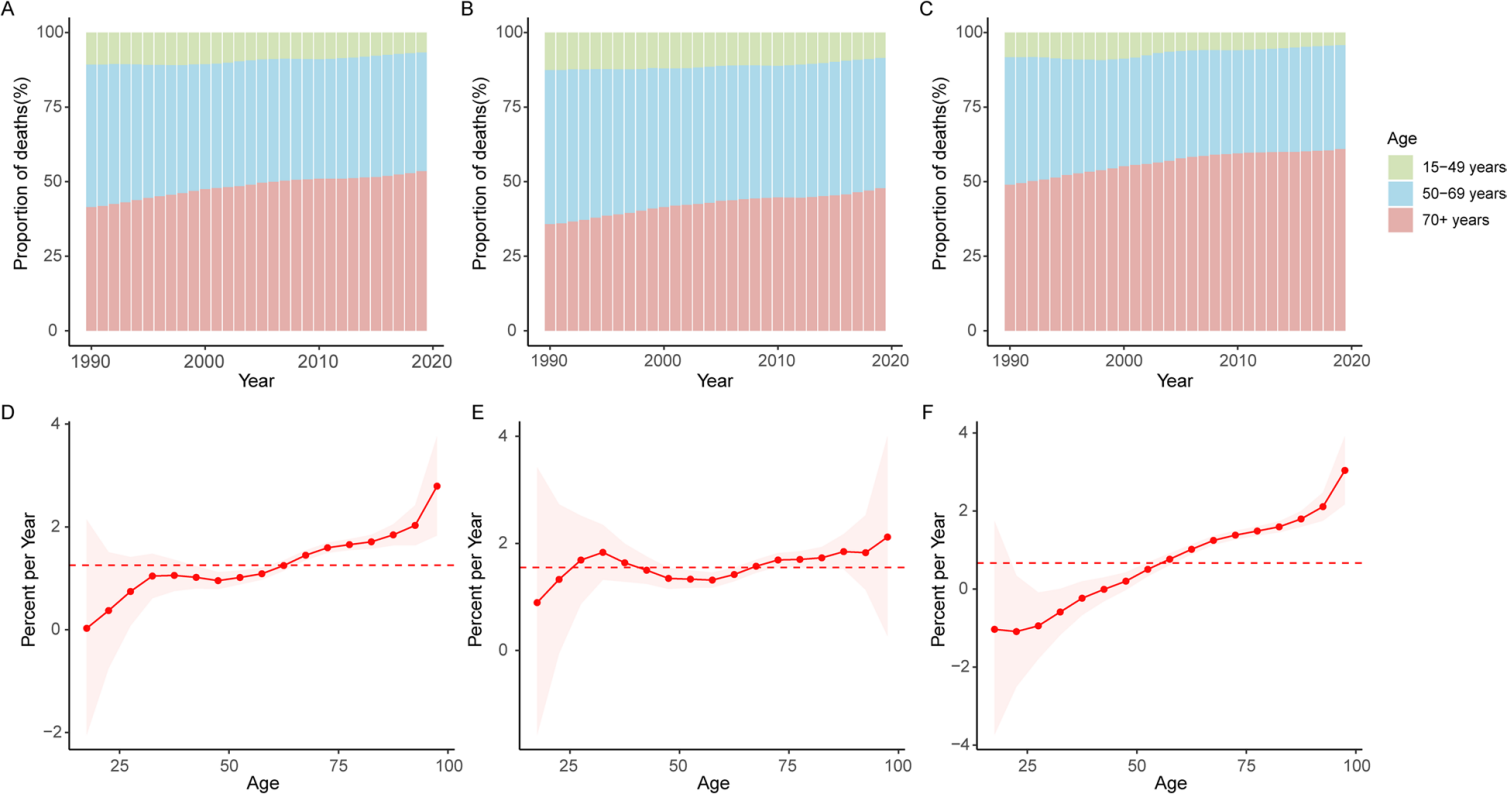
Age distribution of pancreatic cancer deaths and local drifts of pancreatic cancer mortality by SDI and sex, 1990 to 2019. Temporal change in the relative proportion of pancreatic cancer deaths across age groups (15 to 49 years, 50 to 69 years, 70 + years) in (**A**) both sexes, **B** males and (**C**) females, 1990 to 2019. Local drifts of pancreatic cancer mortality (from age-period-cohort models) for 16 age groups in (**D**) both sexes, **E** males and (**F**) females, 1990 to 2019

Figure 5A-C show temporal changes in the age distribution of pancreatic cancer deaths. From 1990 to 2019, people who were over 50 years accounted for the largest proportion of pancreatic cancer deaths in the Western Pacific Region. Additionally, the proportion of people over 70 years old is rising year by year in both sexes. The age distribution of deaths for each country/territory is shown in Fig. S6.

Figure 5D-F show the overall annual percentage change (net drift) and annual percentage change in different age groups (local drifts) of pancreatic cancer mortality. Overall, pancreatic cancer mortality had increasing trends in almost all age groups. The increasing trend was enhanced with increasing age, and death rates increased the most in the oldest group (local drift = 2.79, 95% CI: 1.83 to 3.76). During the study period, net drift for males was higher than that for females. Males over 25 years old had increasing trends for pancreatic cancer death, while females older than 50 years had increasing trends. Additionally, among people over the age of 90, mortality rates increased slower for males than for females. Local drifts and net drifts by sex are shown in Tables S7-S10.

### Age-period-cohort analysis

**Fig. 6:**
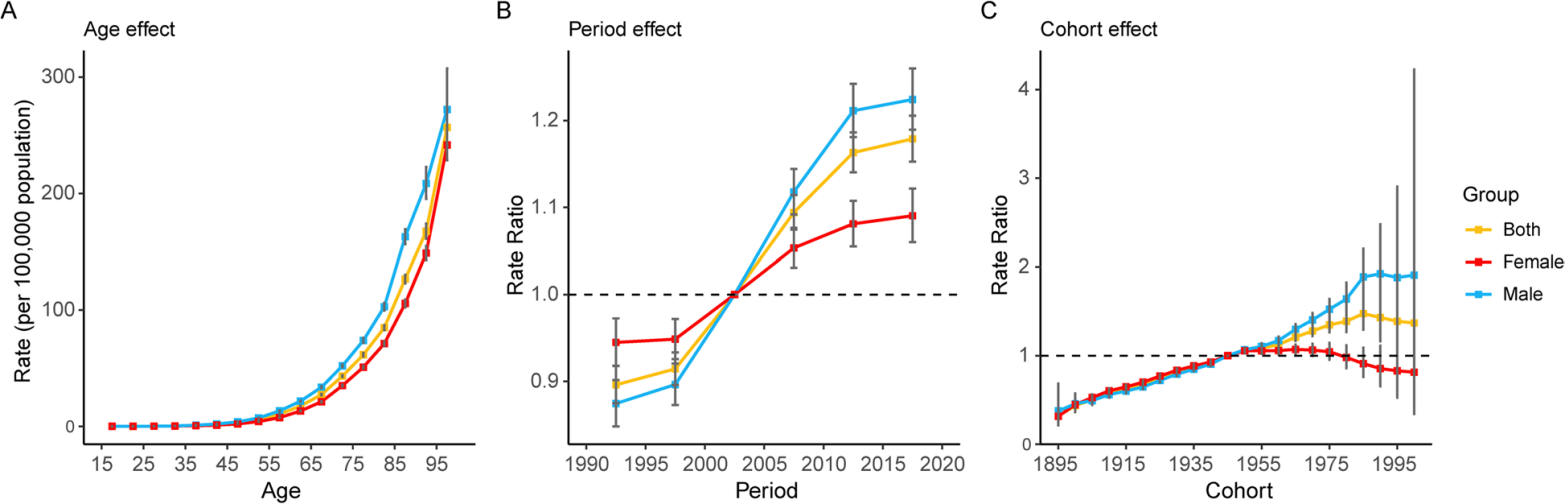
Age, period and cohort effects on pancreatic cancer mortality by sex. **A** Age effects are shown by the fitted longitudinal age curves of mortality (per 100,000). **B** Period effects are shown by the mortality rate ratio. **C** Cohort effects are shown by the mortality rate ratio

Generally, we found similar patterns in age effects in both sexes, with the lowest mortality in young people and with risk increasing with age. The age effect was more pronounced among older men than in older women (aged > 55 years) in the same age group. The period effect showed an increasing risk of mortality for both sexes over the study period. After the 2000 to 2004 period (median: 2002), the mortality risk increased faster in males than in females. Compared to the reference period (2000 to 2004), the pancreatic cancer mortality risk was 1.22 (95% CI: 1.19 to 1.26) in males and 1.09 (95% CI: 1.06 to 1.12) in females in the period of 2015 to 2019. There was an overall increasing rate ratio from early birth cohorts to recent cohorts. Similar to the period effects, the increase in the group effect was more pronounced in males (Fig. 6). The age-period-cohort model on pancreatic cancer mortality in Western Pacific countries/territories is shown in Fig. S7.

### Prediction of mortality to 2044

**Fig. 7:**
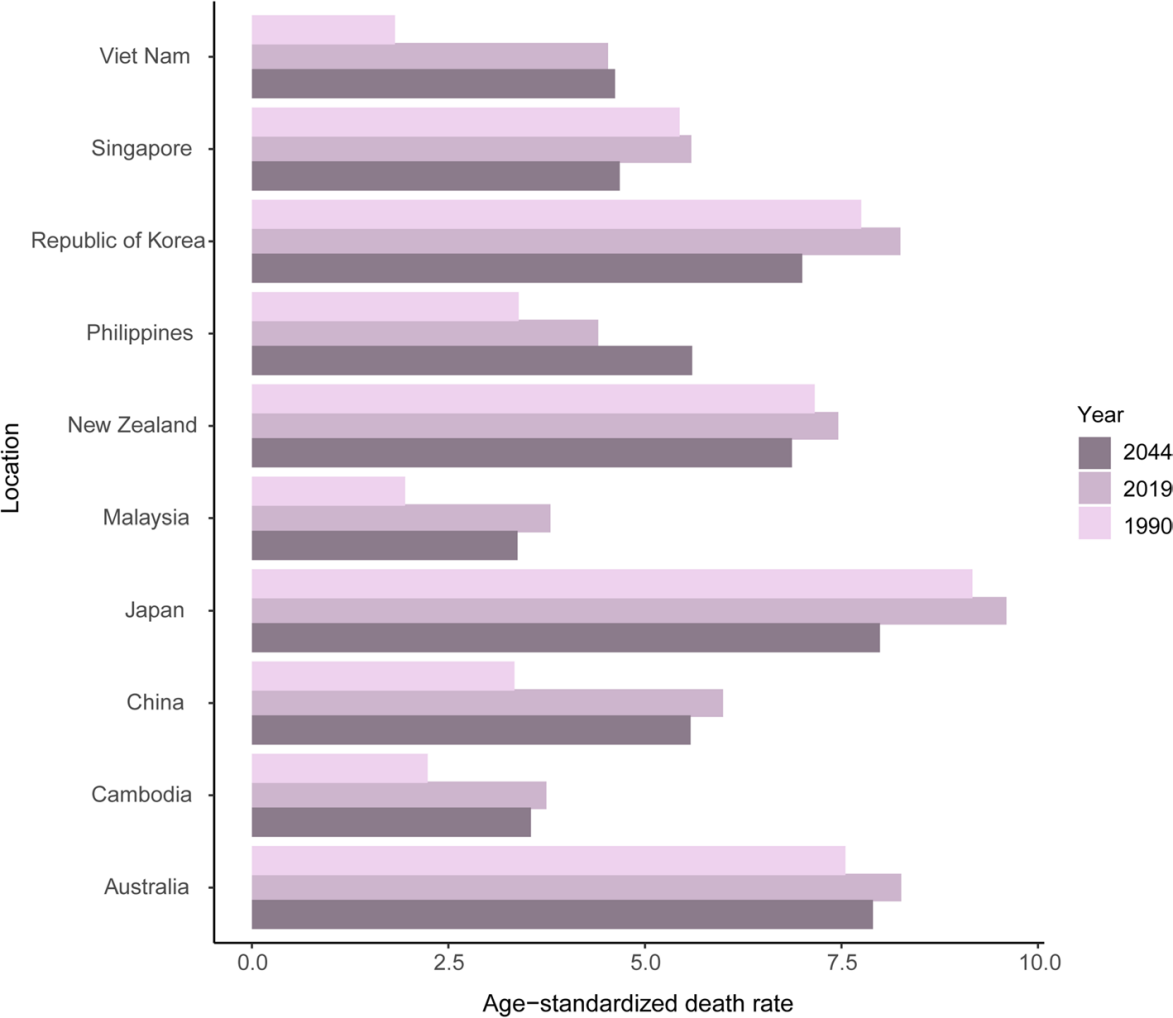
Age-standardized death rates of pancreatic cancer in 1990 and 2019 and predicted rates in 2044 in ten Western Pacific countries

We selected ten countries/territories with the highest pancreatic cancer deaths in 2019 to predict the number and ASR of deaths to 2044, including China, Japan, Republic of Korea, Viet Nam, Australia, Philippines, Malaysia, New Zealand, Singapore and Cambodia (Fig. 7). Most countries have seen their ASDR forecasts fall. ASDRs in 2044 may be 1.27 times higher in 2019 in the Philippines and may slightly increase in Vietnam and Australia. We also predict the number of deaths in these countries from 2020 to 2044 (Fig. S8). The results showed that deaths may continue to increase in the next 25 years in the ten countries.

## Discussion

Cancer is a serious medical and public health problem worldwide, and cancer mortality is becoming an important indicator of progress toward sustainable development goals. In this study, we used an age-period-cohort model to assess the time trends of pancreatic cancer mortality in the Western Pacific Region and predicted the future situation. In 2019, the Western Pacific Region accounted for a high proportion of global pancreatic cancer deaths and may increase in the future, emphasizing the impact of pancreatic cancer burden in this region of the globe. Based on regional situations and global experiences, countries/territories in the Western Pacific Region should develop targeted strategies aimed at high-risk pancreatic cancer populations.

During the past 30 years, the burden of pancreatic cancer was higher in high SDI countries and lower in low SDI countries [12]. Higher pancreatic cancer incidence and mortality in high SDI countries may be due to population aging and lifestyle choices that increase exposure to risk factors, such as obesity and diabetes [12]. For countries with large populations, there were significant increases in DALYs of pancreatic cancer mainly due to population growth and aging. Moreover, high-income countries have great accuracy of cancer-related deaths in databases due to a well-established cancer registry, while low-income and middle-income countries have inadequate access to high-quality cancer data [21–23]. Thus, the differences in the accuracy of data registries influenced the reliability of some analyses in such low- and middle-income countries. The differences in epidemiological accuracy across countries should be given more attention in the future.

Death cases and ASDR of pancreatic cancer were higher in men than in women in the Western Pacific Region over the past 30 years, which was similar to the global level [24]. Females have a lower incidence of pancreatic cancer and may be less likely to be exposed to some risk factors for pancreatic cancer, such as smoking or factors related to hormonal effects [12]. The global age-standardized prevalence of tobacco use in males (32.7%) is nearly five times that in females (6.62%) [25]. Since 1990, some countries in the Western Pacific Region have not had significant reductions in the prevalence of daily smoking among males [26]. The incidence and death of pancreatic cancer in women may be more attributable to metabolic factors. The worldwide prevalence of overweight and obesity was found to be higher in older females than in older males [27, 28]. However, most countries/territories in the Western Pacific Region have a lower prevalence of obesity.

Smoking is a recognized risk factor for various cancers [1, 29, 30]. Despite a significant decline in the prevalence of smoking since 1990, population growth has led to a significant increase in the total number of smokers worldwide [25]. Cigarettes produce a variety of carcinogens, including tobacco-specific nitrosamines, polycyclic aromatic hydrocarbons, and volatile organic compounds [31]. We found that the age-standardized deaths attributable to smoking increased slightly during the past 30 years, and it was still higher than the age-standardized deaths attributable to the two metabolic factors. The Western Pacific Region is home to one-third of the global smoking population, and some countries have a large number of smokers [25, 32]. Despite tobacco control campaigns in many member states, the current rate of tobacco reduction in the Western Pacific is not fast enough to meet the 2025 target [32]. The Framework Convention on Tobacco Control entered into force in 2005, and it redefined approaches to tobacco control and use [33]. Although there has been some regional and national progress in tobacco use and control in recent years, social factors may contribute to the increased disease burden attributable to smoking. We found that population growth and aging are important factors in the increase in pancreatic cancer DALYs, which was similar to the finding from GBD 2015 Tobacco Collaborators: unless progress in reducing tobacco use can be substantially accelerated, population growth is poised to heighten the disease burden associated with smoking [26].

The age effect increased from the youngest to the oldest age group. Several characteristics of aging are very similar to specific cancer hallmarks [34]. Pancreatic cancer has been considered an age-related disease because the cancer appears to share many features with aging, including genomic instability, telomere wear, epigenetic changes, and metabolic alterations [35]. As time goes on, increased exposure to environmental and behavioral factors (such as smoking) may contribute to the chronic accumulation of DNA damage that increases the probability of cancer. Senescent cells in human bodies can manifest as a senescence-associated secretory phenotype, which can secrete various cytokines and growth factors to drive tumorigenesis, including pancreatic cancer [35, 36].

The period effect showed that the mortality risk of pancreatic cancer markedly increased in the Western Pacific Region during the study period. During the past few decades, risk factors for pancreatic cancer have continued to increase among populations. First, the prevalence of smoking has increased; in 2019, male smoking rates were above 20% in more than 150 countries [25, 26]. Among populations over 15 years old, countries with the highest prevalence of tobacco use were mostly in Asia and Oceania [25]. At present, Western Pacific countries are in a critical period of social progress and economic development. People may smoke to relieve pressure and obtain a soothing mood beacuse nicotine causes an acute mood “boost”, including an increased positive effect and a decreased negative effect [37]. Moreover, the westernization of diet and lifestyle in Western Pacific countries may contribute to an increased risk of pancreatic cancer. Dietary patterns changed from a predominantly plant-based diet to a high-energy diet and animal-based foods in some Western Pacific countries [38–40]. The consumption of energy-dense foods such as meat, snacks, and beverages increased, resulting in high BMI and hyperglycemia.

Similar to the period effect, more exposure to risk factors from the early birth cohort to the latest cohort can contribute to pancreatic cancer burden. We noticed that the cohort rate ratio slightly decreased since the cohort was born in 1985 (from 1.47 in 1985 to 1.37 in 2000). In the latest birth cohort, people can receive better science and health education, and access to medical knowledge is also more abundant. Health awareness has improved among younger people, and they may devote more attention to healthy lifestyles and cancer prevention.

We also predict that the number of future deaths from pancreatic cancer in ten Western Pacific countries will continue to increase. This may be related to the increase in population size and change in population structure in the future. The trends of pancreatic cancer mortality across the Western Pacific Region suggested that these countries should implement stronger measures to reduce the disease burden of pancreatic cancer. Countries that had relatively good performance in cancer prevention and reducing mortality can serve as a reference for others, emphasizing primary health care, scientific education and early cancer screening. In some countries where the population continues to grow and the population is aging, more attention will be needed in the health of high-risk populations and early cancer screening. At present, the main treatments for pancreatic cancer include surgery, chemotherapy and immunotherapy [41]. Some new therapeutic strategies, such as chemotherapy combined with immunotherapy, may improve clinical outcomes for pancreatic cancer patients in the future [42].

This study has many limitations. First, the accuracy of the GBD estimates was limited by the quality and availability of each country’s registration system. For some countries/territories without detailed cancer data sources, GBD estimates were mainly generated from modeling processes, neighboring locations and predictive covariates, which may result in potential substantial uncertainty [43]. The reported high mortality in high-income countries might be partly due to robust systems for determining the cause of cancer death and accurate diagnostic data in registration databases. Some low-income countries cannot provide accurate and adequate data on pancreatic cancer mortality due to incomplete health care systems. Thus, the estimates are very likely to substantially underestimate cancer mortality in low-income countries. This hinders the estimates of the actual disease burden in some places. Next, the age-period-cohort model was conducted in a period of five years due to the 5-year intervals in the GBD 2019, which might smoothen some variations in age, period and birth cohort effects. Future work should focus on more primary data on pancreatic cancer mortality, which might include data from registration databases, cancer centers and longer-term cohort studies. This means that more attention should be given to establishing integrative disease surveillance systems to capture pancreatic cancer-related incidence and mortality, especially in developing countries. Considering that the burden of pancreatic cancer varies by age, sex, country and region, the mortality data should also be classified according to more dimensions in the future. The economic burden caused by pancreatic cancer should also be given more attention.

## Conclusion

Pancreatic cancer is an important cause of mortality, and pancreatic cancer deaths continue to increase in the Western Pacific Region. Much of this increase was due to increases in population growth, aging and exposure to risk factors. Our data analysis of pancreatic cancer mortality in Western Pacific Region is limited by the accuracy of data collection in several countries/territories, which needs to be further improved in the future. Modifiable pancreatic cancer risk factors, including behavioral factors and metabolic factors, need to be changed in the Western Pacific region by joint efforts of all countries/territories. Effective cancer prevention approaches and novel anticancer therapy strategies will be essential to achieve the aims of reducing the mortality of pancreatic cancer.

### Supplementary Information

**Additional file 1: Figure S1** The fractions of pancreatic cancer age-specific deaths attributable to smoking, high fasting plasma glucose, and high body mass index by age group by sex, 2019. **Figure S2** The fractions of pancreatic cancer age-standardized deaths attributable to smoking, high fasting plasma glucose, and high body mass index among countries/territories by sex, 2019. **Figure S3 **Decomposition analysis of DALYs attributable to smoking in Western Pacific region, 1990 to 2019. **Figure S4 **Decomposition analysis of DALYs attributable to high fasting plasma glucose in Western Pacific region, 1990 to 2019. **Figure S5 **Decomposition analysis of DALYs attributable to high body mass index in the Western Pacific region, 1990 to 2019. **Figure S6 **Temporal change in the relative proportion of pancreatic cancer deaths across age groups (15 to 49 years, 50 to 69 years, 70+ years) in Western Pacific countries/territories, 1990 to 2019. **Figure S7** Age, period and cohort effects on pancreatic cancer mortality. **Figure S8** Predicting pancreatic cancer deaths to 2044 in ten Western Pacific countries.

**Additional file 2: Table S1 **Rank of pancreatic cancer ASDR in Western Pacific countries for both sexes, 1990 and 2019. **Table S2** Rank of pancreatic cancer ASDR in Western Pacific countries in males, 1990 and 2019. **Table S3** Rank of pancreatic cancer ASDR in Western Pacific countries in females, 1990 and 2019. **Table S4** The deaths and ASDR of pancreatic cancer attributable to smoking in Western Pacific countries/territories in 1990 and 2019. **Table S5** The deaths and ASDR of pancreatic cancer attributable to high fasting plasma glucose in Western Pacific countries/territories in 1990 and 2019. **Table S6** The deaths and ASDR of pancreatic cancer attributable to high body-mass index in Western Pacific countries/territories in 1990 and 2019. **Table S7** Local drifts of pancreatic cancer in the Western Pacific region for both sexes combined, 1990 to 2019. **Table S8** Local drifts of pancreatic cancer in the Western Pacific region for males, 1990 to 2019. **Table S9** Local drifts of pancreatic cancer in the Western Pacific region for females, 1990 to 2019. **Table S10** Net drift of pancreatic cancer in the Western Pacific region, 1990 to 2019.

## Data Availability

The datasets generated and/or analysed during the current study are available in the Global Health Data Exchange query (https://vizhub.healthdata.org/gbd-results/).

## Abbreviations

ASDR: Age-standardized death rate
ASR: Age-standardized rate
CI: Confidence interval
DALY: Disability-adjusted life year
EAPC: Estimated annual percentage change
GBD: Global Burden of Disease
SDI: Sociodemographic index
UI: Uncertainty interval
