# Supplementary material for: Time trend of pancreatic cancer mortality in the Western Pacific Region: age-period-cohort analysis from 1990 to 2019 and forecasting for 2044
Source: BMC Cancer. 2023 Sep 18;23:876. doi: 10.1186/s12885-023-11369-1 (PMC10506228; doi:10.1186/s12885-023-11369-1)

Figure S1 The fractions of pancreatic cancer age-specific deaths attributable to smoking, high fasting plasma glucose, and high body mass index by age group by sex, 2019

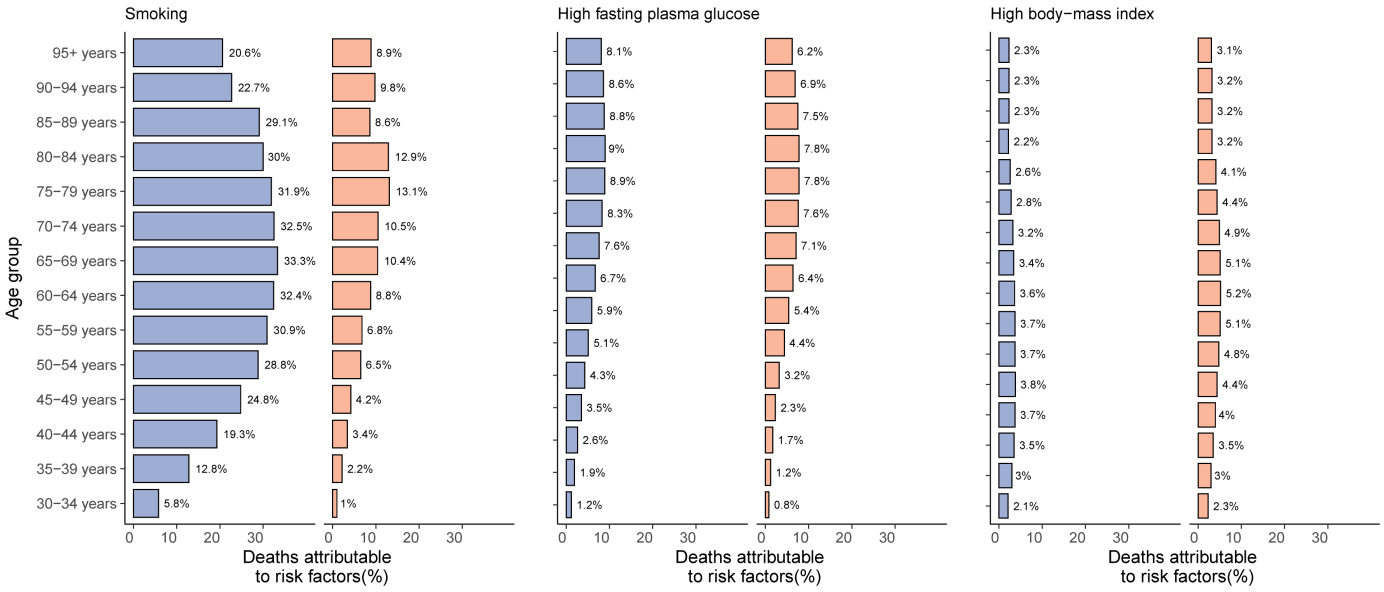

Figure S2 The fractions of pancreatic cancer age-standardized deaths attributable to smoking, high fasting plasma glucose, and high body mass index among countries/territories by sex, 2019

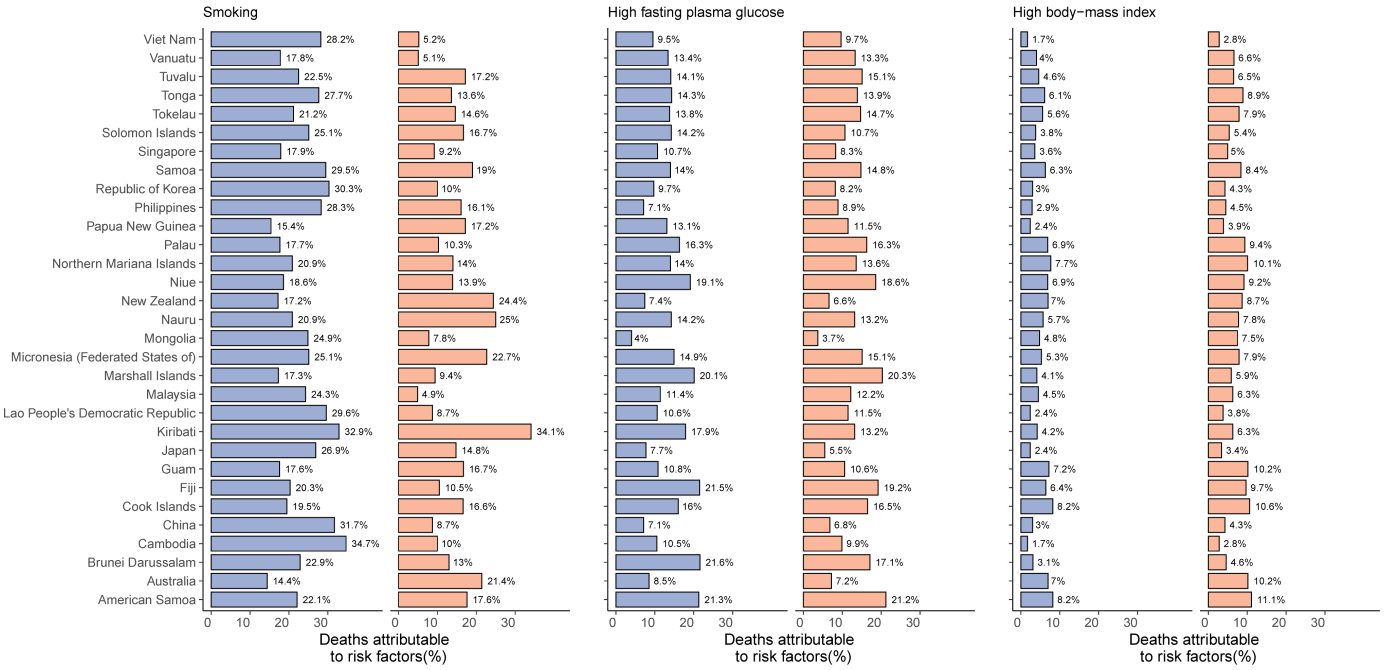

Figure S3 Decomposition analysis of DALYs attributable to smoking in Western Pacific region, 1990 to 2019

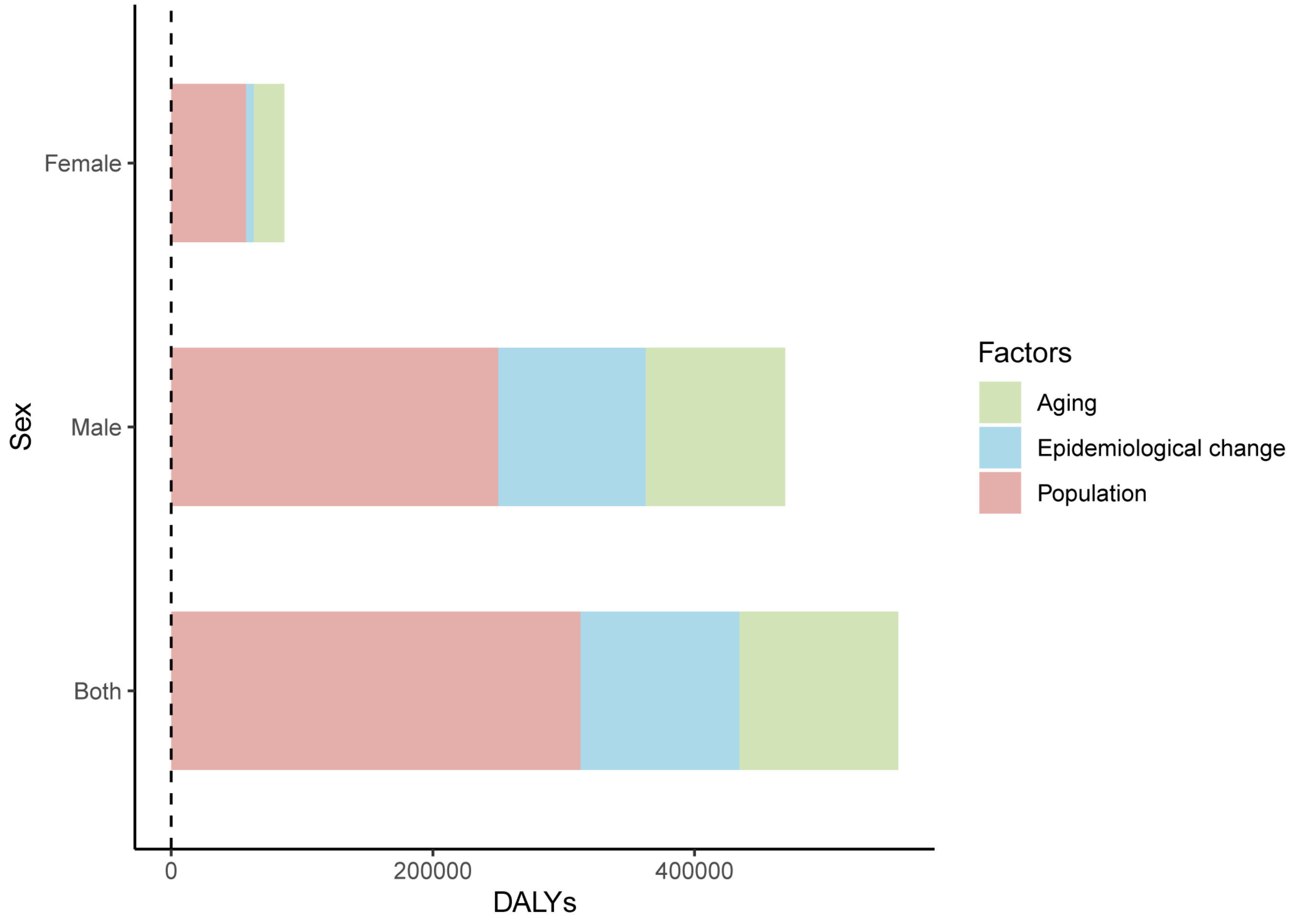

Figure S4 Decomposition analysis of DALYs attributable to high fasting plasma glucose in Western Pacific region, 1990 to 2019

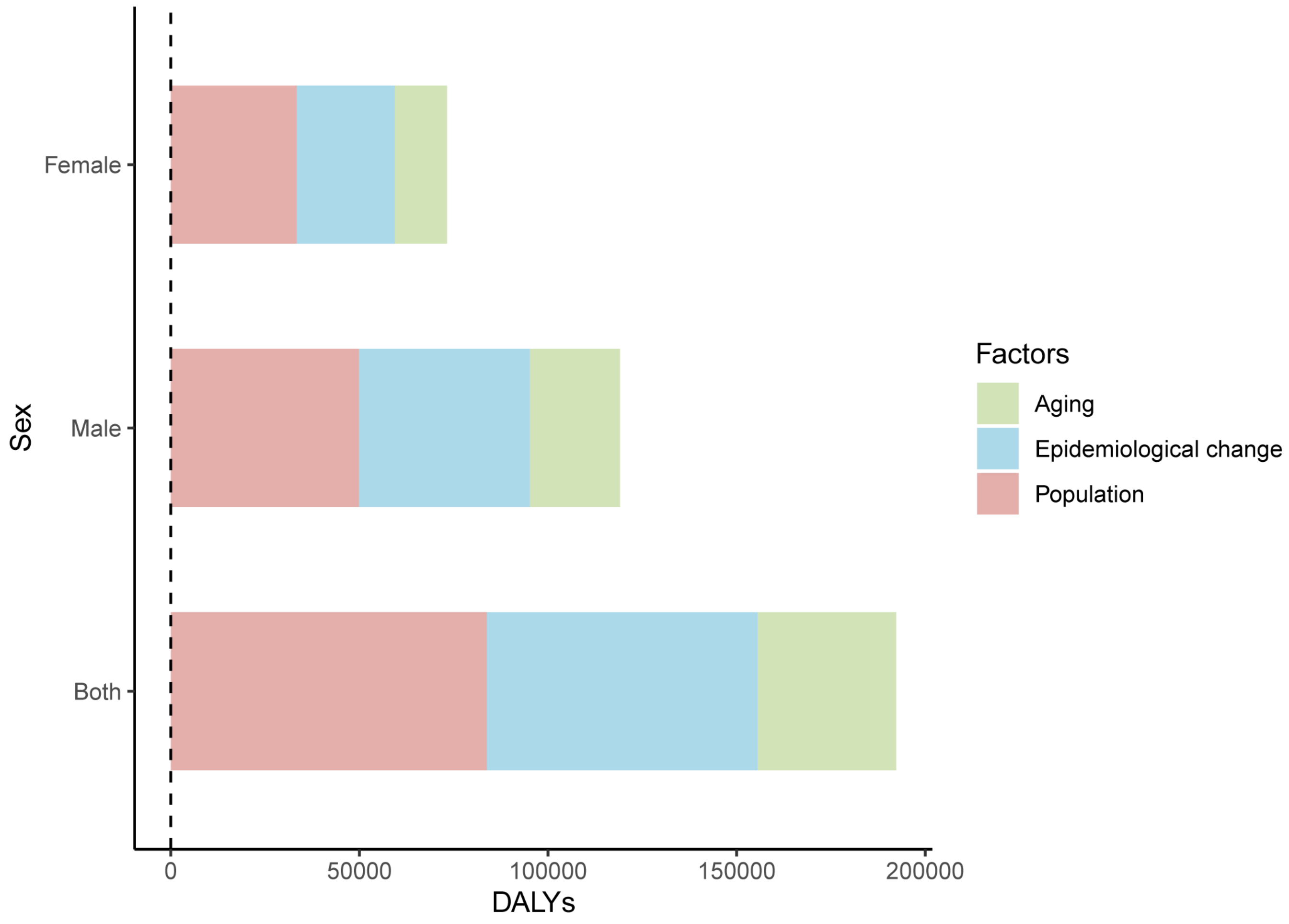

Figure S5 Decomposition analysis of DALYs attributable to high body mass index in Western Pacific region, 1990 to 2019

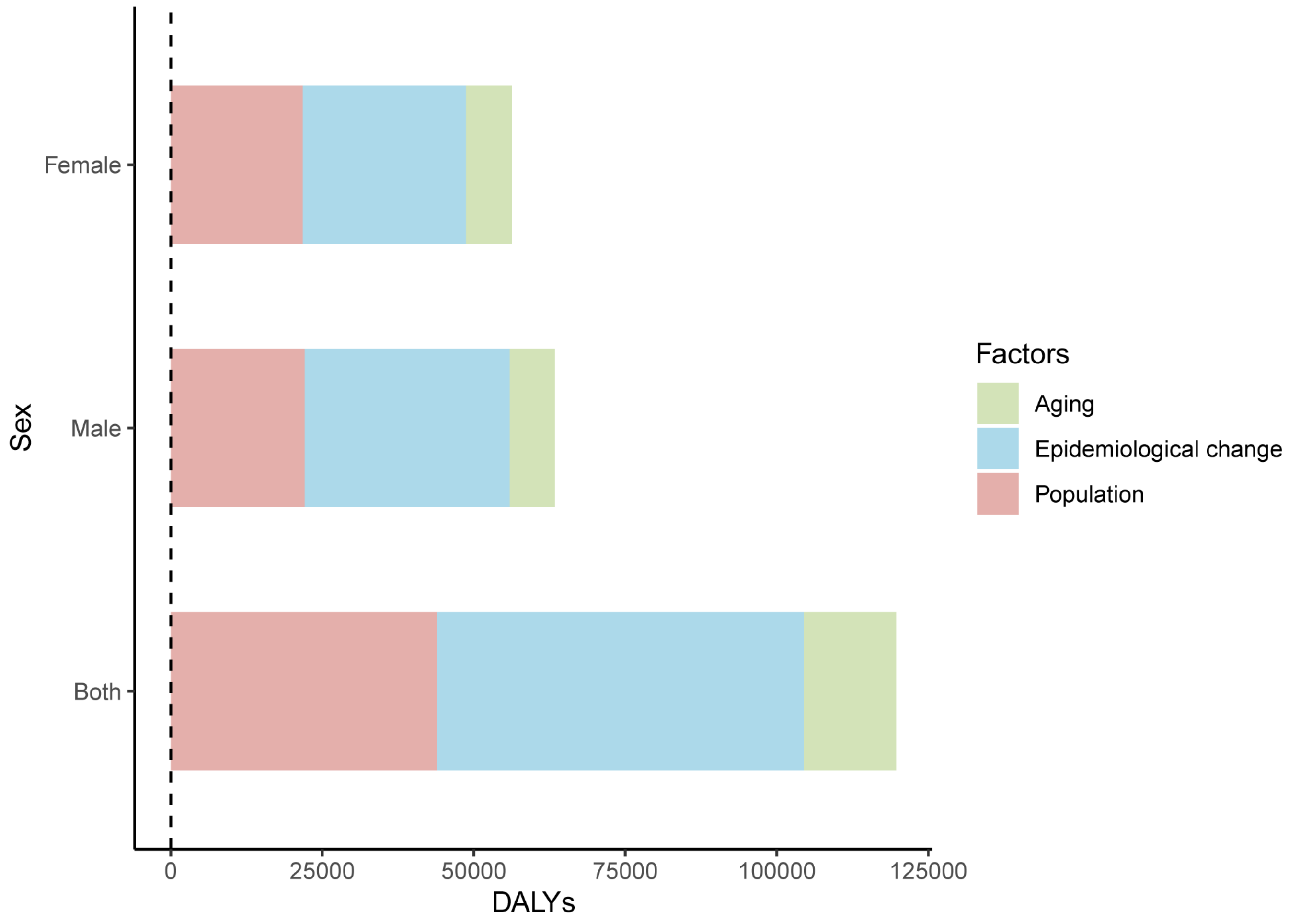

Figure S6 Temporal change in the relative proportion of pancreatic cancer deaths across age groups (15 to 49 year, 50 to 69 years, 70+ years) in Western Pacific countries/territories, 1990 to 2019.

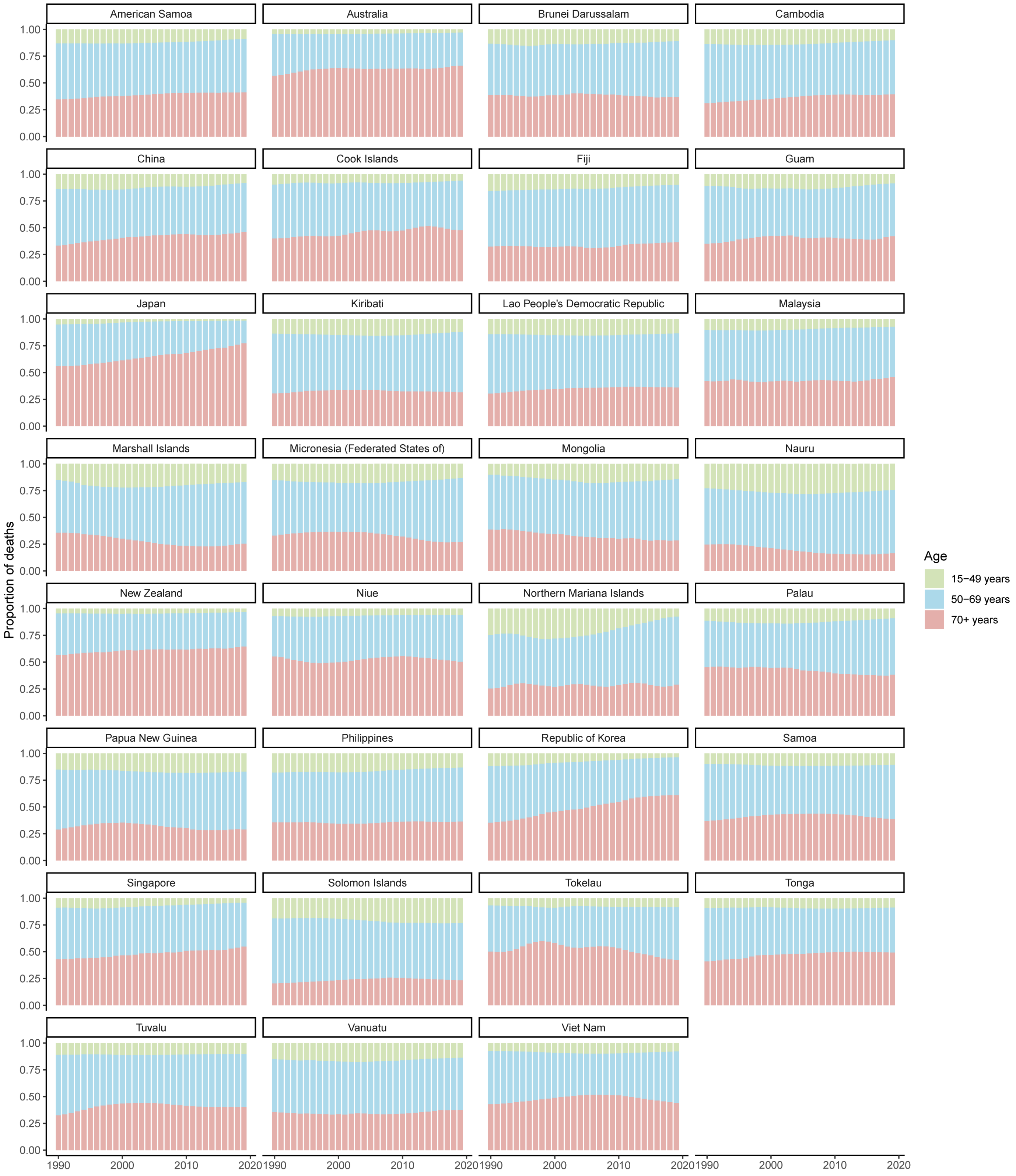

Figure S7 Age, period and cohort effects on pancreatic cancer mortality

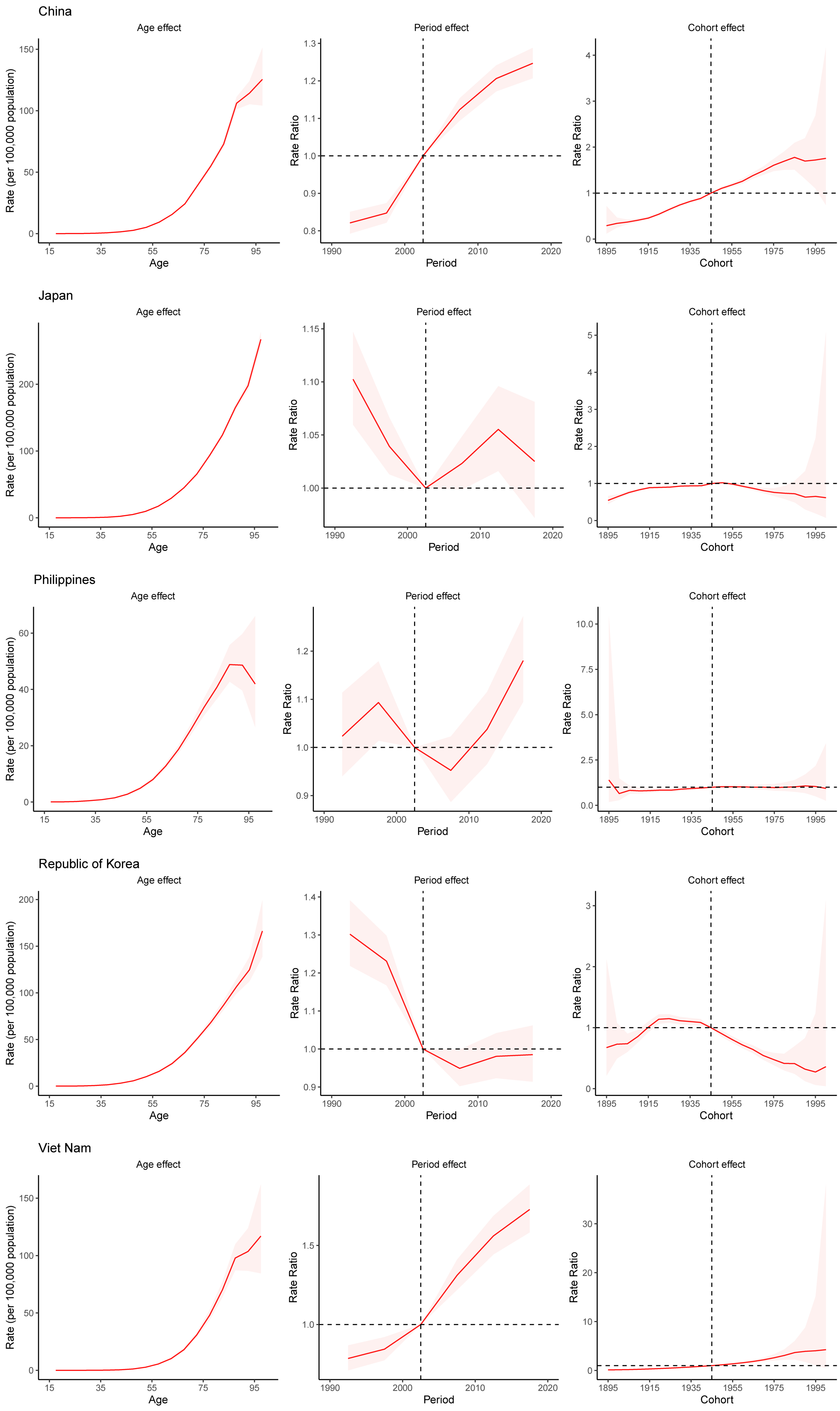

Figure S8 Predicting pancreatic cancer deaths to 2044 in ten Western Pacific countries

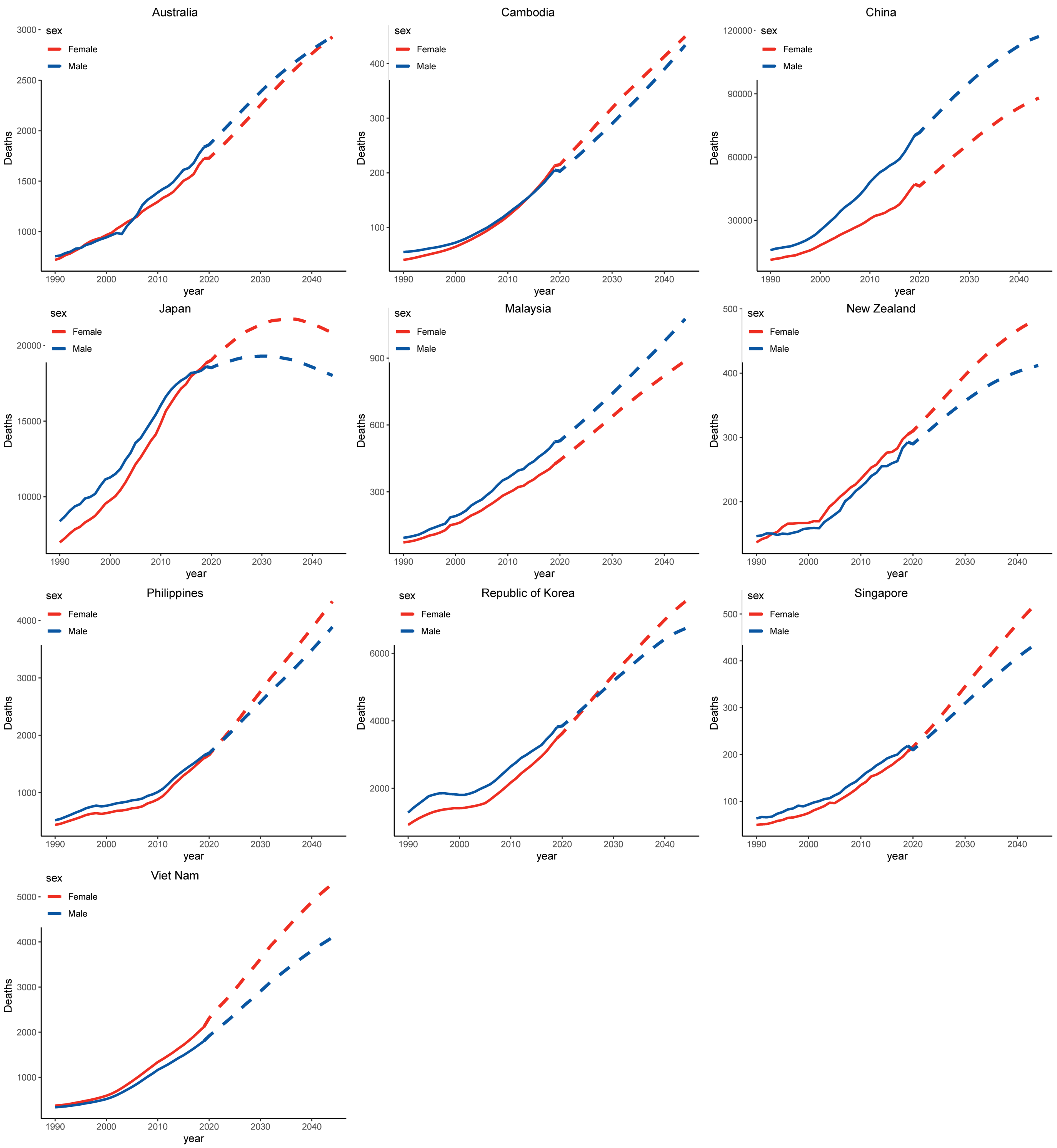

Supplement: Supplementary file 1 — Additional file 1: Figure S1 The fractions of pancreatic cancer age-specific deaths attributable to smoking, high fasting plasma glucose, and high body mass index by age group by sex, 2019. Figure S2 The fractions of pancreatic cancer age-standardized deaths attributable to smoking, high fasting plasma glucose, and high body mass index among countries/territories by sex, 2019. Figure S3 Decomposition analysis of DALYs attributable to smoking in Western Pacific region, 1990 to 2019. Figure S4 Decomposition analysis of DALYs attributable to high fasting plasma glucose in Western Pacific region, 1990 to 2019. Figure S5 Decomposition analysis of DALYs attributable to high body mass index in the Western Pacific region, 1990 to 2019. Figure S6 Temporal change in the relative proportion of pancreatic cancer deaths across age groups (15 to 49 years, 50 to 69 years, 70+ years) in Western Pacific countries/territories, 1990 to 2019. Figure S7 Age, period and cohort effects on pancreatic cancer mortality. Figure S8 Predicting pancreatic cancer deaths to 2044 in ten Western Pacific countries. [file 12885_2023_11369_MOESM1_ESM.pdf]
