# Supplementary material for: Time trend of pancreatic cancer mortality in the Western Pacific Region: age-period-cohort analysis from 1990 to 2019 and forecasting for 2044
Source: BMC Cancer. 2023 Sep 18;23:876. doi: 10.1186/s12885-023-11369-1 (PMC10506228; doi:10.1186/s12885-023-11369-1)
Supplement: Supplementary file 2 — Additional file 2: Table S1 Rank of pancreatic cancer ASDR in Western Pacific countries for both sexes, 1990 and 2019. Table S2 Rank of pancreatic cancer ASDR in Western Pacific countries in males, 1990 and 2019. Table S3 Rank of pancreatic cancer ASDR in Western Pacific countries in females, 1990 and 2019. Table S4 The deaths and ASDR of pancreatic cancer attributable to smoking in Western Pacific countries/territories in 1990 and 2019. Table S5 The deaths and ASDR of pancreatic cancer attributable to high fasting plasma glucose in Western Pacific countries/territories in 1990 and 2019. Table S6 The deaths and ASDR of pancreatic cancer attributable to high body-mass index in Western Pacific countries/territories in 1990 and 2019. Table S7 Local drifts of pancreatic cancer in the Western Pacific region for both sexes combined, 1990 to 2019. Table S8 Local drifts of pancreatic cancer in the Western Pacific region for males, 1990 to 2019. Table S9 Local drifts of pancreatic cancer in the Western Pacific region for females, 1990 to 2019. Table S10 Net drift of pancreatic cancer in the Western Pacific region, 1990 to 2019. [file 12885_2023_11369_MOESM2_ESM.pdf]

Additional File 2: Supplementary tables

Table S1 Rank of pancreatic cancer ASDR in Western Pacific countries for both sex, 1990 and 2019

Country	ASDR in 1990	Rank	ASDR in 2019	Rank
Japan	9.17	1	9.60	2
Palau	8.38	2	11.95	1
Republic of Korea	7.74	3	8.25	5
Australia	7.55	4	8.26	4
New Zealand	7.16	5	7.46	7
Brunei Darussalam	6.62	6	9.30	3
Singapore	5.44	7	5.59	12
Nauru	4.35	8	6.08	10
Guam	4.28	9	5.26	14
Samoa	4.26	10	4.40	21
Niue	3.87	11	6.40	8
Cook Islands	3.80	12	4.95	16
Northern Mariana Islands	3.55	13	5.59	6
Philippines	3.39	14	4.41	20
China	3.34	15	5.99	11
American Samoa	3.26	16	5.19	15
Micronesia (Federated States of)	3.24	17	5.31	13
Mongolia	3.23	18	6.28	9
Tonga	2.96	19	4.49	18
Fiji	2.74	20	4.34	22

Tokelau	2.65	21	4.46	19
Tuvalu	2.62	22	4.02	23
Marshall Islands	2.42	23	4.01	24
Lao People's Democratic Republic	2.40	24	3.52	27
Kiribati	2.37	25	3.00	30
Cambodia	2.23	26	3.75	26
Malaysia	1.95	27	3.80	25
Vanuatu	1.91	28	3.11	28
Solomon Islands	1.87	29	3.07	29
Viet Nam	1.82	30	4.53	17
Papua New Guinea	1.26	31	1.81	31

ASDR: age-standardized rate

Table S2 Rank of pancreatic cancer ASDR in Western Pacific countries in male, 1990 and 2019

Country	ASDR in 1990	Rank	ASDR in 2019	Rank
Japan	11.75	1	11.55	1
Republic of Korea	10.80	2	9.91	3
Australia	8.84	3	9.36	4
New Zealand	8.51	4	8.02	6
Brunei Darussalam	8.16	5	10.40	2
Singapore	6.73	6	6.08	14
Samoa	5.34	7	5.17	18
Cook Islands	5.24	8	6.65	12
Nauru	4.79	9	7.00	10
Guam	4.65	10	6.07	15
Niue	4.26	11	7.31	9
American Samoa	4.21	12	6.78	11
China	4.12	13	7.69	7
Tonga	4.02	14	6.43	13
Mongolia	3.88	15	8.4	5
Micronesia (Federated States of)	3.67	16	5.87	16
Fiji	3.62	17	5.68	17
Philippines	3.62	18	4.72	20
Northern Mariana Islands	3.44	19	7.64	8
Lao People's Democratic Republic	3.00	20	3.74	27
Cambodia	2.98	21	4.43	21
Tuvalu	2.97	22	4.33	22
Marshall Islands	2.91	23	4.32	23
Kiribati	2.58	24	3.22	30

Tokelau	2.43	25	4.12	25
Palau	2.41	26	3.83	26
Solomon Islands	2.28	27	3.66	28
Malaysia	2.23	28	4.15	24
Vanuatu	2.19	29	3.55	29
Viet Nam	2.07	30	4.87	19
Papua New Guinea	1.52	31	2.11	31

ASDR: age-standardized rate

Table S3 Rank of pancreatic cancer ASDR in Western Pacific countries in female, 2019

Country	ASDR in 2019	Rank	ASDR in 2019	Rank
Palau	13.91	1	19.34	1
Japan	7.18	2	7.85	3
Australia	6.47	3	7.24	5
New Zealand	6.07	4	6.95	6
Republic of Korea	5.71	5	6.84	7
Brunei Darussalam	5.55	6	8.88	2
Singapore	4.43	7	5.14	10
Nauru	3.89	8	5.30	9
Guam	3.85	9	4.45	15
Northern Mariana Islands	3.64	10	7.85	4
Niue	3.53	11	5.60	8
Samoa	3.25	12	3.66	21
Philippines	3.17	13	4.09	17
Tokelau	2.82	14	4.77	13
Micronesia (Federated States of)	2.81	15	4.78	12
Mongolia	2.76	16	4.79	11
China	2.70	17	4.58	14
Tuvalu	2.35	18	3.73	18
American Samoa	2.30	19	3.70	19
Cook Islands	2.23	20	3.37	23
Kiribati	2.21	21	2.86	27
Marshall Islands	1.96	22	3.70	20
Tonga	1.95	23	2.83	28
Fiji	1.89	24	3.27	26

Lao People's Democratic Republic	1.88	25	3.32	24
Malaysia	1.69	26	3.44	22
Cambodia	1.68	27	3.27	25
Viet Nam	1.63	28	4.23	16
Vanuatu	1.57	29	2.63	29
Solomon Islands	1.34	30	2.45	30
Papua New Guinea	1.00	31	1.48	31

ASDR: age-standardized rate

Table S4 The deaths and ASDR of pancreatic cancer attributable to smoking in Western Pacific countries/territories in 1990 and 2019.

Location	Deaths in 1990	ASDR in 1990	Deaths in 2019	ASDR in 2019
American Samoa	0 (0-0)	0.73 (0.55-0.93)	0 (0-1)	1.05 (0.81-1.35)
Australia	432 (376-487)	2.18 (1.89-2.46)	615 (514-734)	1.47 (1.24-1.74)
Brunei Darussalam	1 (1-2)	1.78 (1.41-2.24)	4 (3-5)	1.63 (1.26-2.03)
Cambodia	23 (15-33)	0.58 (0.38-0.81)	86 (65-106)	0.81 (0.62-0.97)
China	5529 (4381-6837)	0.69 (0.56-0.84)	26552 (20856-33174)	1.34 (1.05-1.65)
Cook Islands	0 (0-0)	0.8 (0.59-1.06)	0 (0-0)	0.91 (0.68-1.19)
Fiji	2 (2-3)	0.67 (0.5-0.86)	5 (3-7)	0.7 (0.5-0.98)
Guam	1 (0-1)	0.74 (0.55-0.95)	2 (1-2)	0.9 (0.68-1.18)
Japan	4759 (4200-5379)	2.78 (2.45-3.14)	7434 (6196-8628)	2.04 (1.75-2.34)
Kiribati	0 (0-0)	0.74 (0.58-0.92)	1 (0-1)	1.01 (0.75-1.33)
Lao People's Democratic Republic	12 (7-18)	0.59 (0.38-0.9)	26 (19-35)	0.67 (0.49-0.87)
Malaysia	30 (23-38)	0.36 (0.28-0.45)	145 (104-199)	0.59 (0.42-0.78)
Marshall Islands	0 (0-0)	0.36 (0.26-0.47)	0 (0-0)	0.55 (0.39-0.76)
Micronesia (Federated States of)	0 (0-0)	0.8 (0.58-1.06)	1 (1-1)	1.26 (0.81-1.73)
Mongolia	5 (4-7)	0.52 (0.38-0.66)	24 (17-32)	1.08 (0.8-1.42)
Nauru	0 (0-0)	1.04 (0.73-1.44)	0 (0-0)	1.41 (0.92-1.98)
New Zealand	90 (78-103)	2.26 (1.95-2.58)	124 (103-149)	1.55 (1.29-1.86)
Niue	0 (0-0)	0.69 (0.49-0.93)	0 (0-0)	1.03 (0.73-1.43)
Northern Mariana Islands	0 (0-0)	0.73 (0.56-0.95)	1 (1-1)	1.34 (1.01-1.69)
Palau	0 (0-0)	1.11 (0.76-1.58)	0 (0-0)	1.36 (0.93-1.89)
Papua New Guinea	4 (2-7)	0.24 (0.14-0.38)	13 (8-20)	0.29 (0.18-0.44)
Philippines	224 (180-271)	0.83 (0.68-0.99)	689 (525-882)	0.97 (0.76-1.23)
Republic of Korea	581 (502-661)	2.02 (1.73-2.31)	1510 (1249-1784)	1.7 (1.4-2.01)
Samoa	1 (1-1)	1.08 (0.86-1.35)	2 (1-2)	1.09 (0.83-1.47)

Singapore	23 (19-27)	1.09 (0.91-1.29)	59 (49-70)	0.76 (0.63-0.91)
Solomon Islands	1 (0-1)	0.46 (0.31-0.65)	2 (1-3)	0.67 (0.46-0.92)
Tokelau	0 (0-0)	0.51 (0.35-0.7)	0 (0-0)	0.78 (0.51-1.08)
Tonga	0 (0-1)	0.77 (0.54-1.03)	1 (1-1)	1.02 (0.7-1.41)
Tuvalu	0 (0-0)	0.56 (0.41-0.73)	0 (0-0)	0.79 (0.55-1.1)
Vanuatu	0 (0-0)	0.32 (0.22-0.46)	1 (0-1)	0.4 (0.27-0.55)
Viet Nam	115 (84-149)	0.29 (0.22-0.38)	606 (439-822)	0.69 (0.51-0.93)
Western Pacific Region	11945 (10400-13605)	1.1 (0.97-1.25)	38359 (31806-45750)	1.42 (1.18-1.67)

ASDR: age-standardized death rate.

Table S5 The deaths and ASDR of pancreatic cancer attributable to high fasting plasma glucose in Western Pacific countries/territories in 1990 and 2019.

Location	Deaths in 1990	ASDR in 1990	Deaths in 2019	ASDR in 2019
American Samoa	0 (0-0)	0.51 (0.12-1.06)	0 (0-1)	1.1 (0.29-2.23)
Australia	74 (17-166)	0.38 (0.09-0.85)	294 (66-649)	0.65 (0.15-1.44)
Brunei Darussalam	1 (0-2)	1.18 (0.3-2.41)	4 (1-8)	1.77 (0.44-3.57)
Cambodia	4 (1-9)	0.1 (0.02-0.24)	39 (9-85)	0.38 (0.09-0.82)
China	1523 (323-3532)	0.2 (0.04-0.46)	8046 (1759-18320)	0.41 (0.09-0.94)
Cook Islands	0 (0-0)	0.4 (0.09-0.88)	0 (0-0)	0.8 (0.19-1.68)
Fiji	1 (0-2)	0.37 (0.09-0.81)	6 (1-12)	0.88 (0.23-1.8)
Guam	0 (0-0)	0.35 (0.08-0.79)	1 (0-2)	0.56 (0.13-1.22)
Japan	931 (205-2079)	0.55 (0.12-1.24)	2628 (581-5869)	0.64 (0.14-1.43)
Kiribati	0 (0-0)	0.23 (0.05-0.51)	0 (0-1)	0.45 (0.11-1)
Lao People's Democratic Republic	3 (1-7)	0.15 (0.03-0.39)	14 (3-31)	0.39 (0.09-0.84)
Malaysia	13 (3-28)	0.16 (0.03-0.36)	105 (25-233)	0.45 (0.11-0.98)
Marshall Islands	0 (0-0)	0.34 (0.08-0.74)	0 (0-1)	0.81 (0.21-1.72)
Micronesia (Federated States of)	0 (0-0)	0.26 (0.06-0.59)	0 (0-1)	0.79 (0.2-1.74)
Mongolia	1 (0-2)	0.08 (0.02-0.19)	5 (1-11)	0.24 (0.05-0.57)
Nauru	0 (0-0)	0.38 (0.08-0.86)	0 (0-0)	0.84 (0.19-1.91)
New Zealand	13 (3-28)	0.32 (0.07-0.71)	44 (10-96)	0.52 (0.12-1.15)
Niue	0 (0-0)	0.47 (0.11-1.03)	0 (0-0)	1.21 (0.3-2.58)
Northern Mariana Islands	0 (0-0)	0.32 (0.07-0.71)	0 (0-1)	1.07 (0.27-2.23)
Palau	0 (0-0)	0.87 (0.17-2.02)	0 (0-1)	1.96 (0.43-4.36)
Papua New Guinea	2 (0-5)	0.11 (0.02-0.29)	9 (2-23)	0.23 (0.05-0.56)
Philippines	56 (13-126)	0.23 (0.05-0.51)	238 (53-524)	0.36 (0.08-0.78)
Republic of Korea	134 (29-298)	0.5 (0.11-1.1)	657 (150-1434)	0.74 (0.17-1.61)
Samoa	0 (0-1)	0.41 (0.09-0.94)	1 (0-2)	0.63 (0.15-1.38)

Singapore	10 (2-22)	0.51 (0.12-1.1)	40 (9-88)	0.53 (0.12-1.18)
Solomon Islands	0 (0-0)	0.13 (0.03-0.33)	1 (0-3)	0.39 (0.09-0.89)
Tokelau	0 (0-0)	0.24 (0.06-0.55)	0 (0-0)	0.64 (0.15-1.43)
Tonga	0 (0-0)	0.28 (0.06-0.65)	0 (0-1)	0.63 (0.14-1.4)
Tuvalu	0 (0-0)	0.23 (0.05-0.53)	0 (0-0)	0.59 (0.13-1.33)
Vanuatu	0 (0-0)	0.16 (0.03-0.38)	1 (0-2)	0.42 (0.1-0.97)
Viet Nam	37 (8-86)	0.1 (0.02-0.23)	344 (81-776)	0.43 (0.1-0.97)
Western Pacific Region	2828 (610-6322)	0.27 (0.06-0.61)	12738 (2881-28589)	0.48 (0.11-1.08)

ASDR: age-standardized death rate.

Table S6 The deaths and ASDR of pancreatic cancer attributable to high body-mass index in Western Pacific countries/territories in 1990 and 2019.

Location	Deaths in 1990	ASDR in 1990	Deaths in 2019	ASDR in 2019
American Samoa	0 (0-0)	0.28 (0.09-0.53)	0 (0-0)	0.48 (0.16-0.91)
Australia	94 (34-178)	0.48 (0.17-0.91)	300 (115-537)	0.71 (0.27-1.26)
Brunei Darussalam	0 (0-0)	0.14 (0.03-0.37)	1 (0-2)	0.37 (0.12-0.76)
Cambodia	1 (0-3)	0.02 (0-0.06)	10 (3-22)	0.09 (0.03-0.19)
China	456 (78-1231)	0.06 (0.01-0.15)	4236 (1108-9602)	0.21 (0.06-0.48)
Cook Islands	0 (0-0)	0.26 (0.07-0.54)	0 (0-0)	0.45 (0.13-0.86)
Fiji	1 (0-1)	0.15 (0.05-0.32)	2 (1-5)	0.34 (0.12-0.65)
Guam	0 (0-0)	0.29 (0.1-0.57)	1 (0-2)	0.45 (0.16-0.85)
Japan	392 (91-936)	0.23 (0.05-0.56)	1025 (252-2417)	0.27 (0.07-0.63)
Kiribati	0 (0-0)	0.12 (0.04-0.24)	0 (0-0)	0.16 (0.05-0.33)
Lao People's Democratic Republic	1 (0-2)	0.03 (0.01-0.07)	5 (1-10)	0.11 (0.03-0.23)
Malaysia	5 (2-11)	0.06 (0.02-0.12)	52 (19-105)	0.2 (0.07-0.4)
Marshall Islands	0 (0-0)	0.09 (0.02-0.2)	0 (0-0)	0.2 (0.06-0.42)
Micronesia (Federated States of)	0 (0-0)	0.18 (0.06-0.37)	0 (0-1)	0.35 (0.13-0.69)
Mongolia	2 (1-3)	0.15 (0.05-0.3)	9 (3-17)	0.38 (0.12-0.73)
Nauru	0 (0-0)	0.27 (0.09-0.56)	0 (0-0)	0.41 (0.13-0.83)
New Zealand	17 (6-32)	0.42 (0.15-0.82)	47 (17-85)	0.59 (0.21-1.08)
Niue	0 (0-0)	0.25 (0.09-0.47)	0 (0-0)	0.52 (0.18-1.01)
Northern Mariana Islands	0 (0-0)	0.32 (0.12-0.59)	0 (0-1)	0.69 (0.26-1.27)
Palau	0 (0-0)	0.65 (0.23-1.21)	0 (0-0)	1.06 (0.4-1.93)
Papua New Guinea	1 (0-2)	0.03 (0.01-0.09)	3 (1-7)	0.05 (0.01-0.14)
Philippines	22 (6-48)	0.07 (0.02-0.16)	126 (42-252)	0.17 (0.06-0.33)
Republic of Korea	58 (14-141)	0.2 (0.05-0.49)	270 (78-587)	0.3 (0.09-0.66)
Samoa	0 (0-1)	0.3 (0.1-0.58)	0 (0-1)	0.32 (0.1-0.63)

Singapore	3 (1-7)	0.13 (0.03-0.32)	19 (6-38)	0.24 (0.08-0.49)
Solomon Islands	0 (0-0)	0.06 (0.01-0.15)	0 (0-1)	0.14 (0.04-0.31)
Tokelau	0 (0-0)	0.13 (0.04-0.26)	0 (0-0)	0.3 (0.11-0.59)
Tonga	0 (0-0)	0.17 (0.05-0.36)	0 (0-1)	0.32 (0.09-0.66)
Tuvalu	0 (0-0)	0.1 (0.03-0.23)	0 (0-0)	0.22 (0.07-0.45)
Vanuatu	0 (0-0)	0.07 (0.02-0.17)	0 (0-1)	0.16 (0.05-0.34)
Viet Nam	6 (1-17)	0.01 (0-0.04)	94 (28-206)	0.11 (0.03-0.23)
Western Pacific Region	1067 (244-2636)	0.1 (0.02-0.24)	6312 (1813-14033)	0.23 (0.07-0.52)

ASDR: age-standardized death rate.

Table S7 Local drifts of pancreatic cancer in Western Pacific region for both sexes combined, 1990 to 2019

Age	Percent per year	Lower 95% CI	Upper 95% CI
17.5	0.04	-2.04	2.15
22.5	0.37	-0.75	1.51
27.5	0.74	0.07	1.41
32.5	1.04	0.61	1.47
37.5	1.06	0.75	1.36
42.5	1.02	0.80	1.24
47.5	0.95	0.79	1.12
52.5	1.02	0.88	1.15
57.5	1.09	0.97	1.20
62.5	1.25	1.15	1.35
67.5	1.45	1.35	1.54
72.5	1.59	1.50	1.69
77.5	1.66	1.55	1.76
82.5	1.71	1.57	1.85
87.5	1.84	1.64	2.05
92.5	2.03	1.64	2.42
97.5	2.79	1.83	3.76

CI: confidence interval.

Table S8 Local drifts of pancreatic cancer in Western Pacific region for males, 1990 to 2019

Age	Percent per year	Lower 95% CI	Upper 95% CI
17.5	0.89	-1.57	3.42
22.5	1.33	-0.06	2.73
27.5	1.69	0.87	2.52
32.5	1.83	1.32	2.35
37.5	1.64	1.28	1.99
42.5	1.50	1.24	1.76
47.5	1.35	1.15	1.54
52.5	1.33	1.17	1.50
57.5	1.32	1.17	1.46
62.5	1.42	1.29	1.55
67.5	1.57	1.45	1.70
72.5	1.69	1.56	1.82
77.5	1.70	1.55	1.85
82.5	1.73	1.53	1.93
87.5	1.85	1.52	2.18
92.5	1.83	1.14	2.53
97.5	2.12	0.87	3.42

CI: confidence interval.

Table S9 Local drifts of pancreatic cancer in Western Pacific region for females, 1990 to 2019

Age	Percent per year	Lower 95% CI	Upper 95% CI
17.5	-1.03	-3.74	1.74
22.5	-1.09	-2.5	0.34
27.5	-0.94	-1.79	-0.09
32.5	-0.59	-1.18	0.00
37.5	-0.23	-0.66	0.20
42.5	0.00	-0.31	0.30
47.5	0.20	-0.02	0.43
52.5	0.50	0.33	0.68
57.5	0.76	0.61	0.91
62.5	1.02	0.89	1.14
67.5	1.25	1.13	1.36
72.5	1.38	1.27	1.49
77.5	1.49	1.37	1.61
82.5	1.59	1.45	1.73
87.5	1.79	1.59	2.00
92.5	2.11	1.75	2.48
97.5	3.04	2.17	3.92

CI: confidence interval.

Table S10 Net drift of pancreatic cancer in Western Pacific region, 1990 to 2019

Net Drift (%/year)	Lower 95% CI	Upper 95% CI	Group
1.255	1.3	1.38	Both
1.553	1.394	1.712	Male
0.663	0.502	0.825	Female

CI: confidence interval.
